# Rational modification of tricarboxylic acid cycle for improving l-lysine production in *Corynebacterium glutamicum*

**DOI:** 10.1186/s12934-018-0958-z

**Published:** 2018-07-07

**Authors:** Jian-Zhong Xu, Ze-Hua Wu, Shi-Jun Gao, Weiguo Zhang

**Affiliations:** 1 The Key Laboratory of Industrial Biotechnology, Ministry of Education, School of Biotechnology, Jiangnan University, 1800# Lihu Road, Wuxi, 214122 People’s Republic of China; 2 Research and Development Department, Shandong Shouguang Juneng Golden Corn Co., Ltd., 1199# Xinxing Street, Shouguang, 262700 People’s Republic of China

**Keywords:** *Corynebacterium glutamicum*, l-Lysine production, Phosphoenolpyruvate–pyruvate–oxaloacetate node, Tricarboxylate synthase, Glutamate dehydrogenase, Biotin

## Abstract

**Background:**

Oxaloacetate (OAA) and l-glutamate are essential precursors for the biosynthesis of l-lysine. Reasonable control of all potentially rate-limiting steps, including the precursors supply rate, is of vital importance to maximize the efficiency of l-lysine fermentation process.

**Results:**

In this paper, we have rationally engineered the tricarboxylic acid (TCA) cycle that increased the carbon yield (from 36.18 to 59.65%), final titer (from 14.47 ± 0.41 to 23.86 ± 2.16 g L^−1^) and productivity (from 0.30 to 0.50 g L^−1^ h^−1^) of l-lysine by *Corynebacterium glutamicum* in shake-flask fermentation because of improving the OAA and l-glutamate availability. To do this, the phosphoenolpyruvate–pyruvate–oxaloacetate (PEP–pyruvate–OAA) node’s genes *ppc* and *pyc* were inserted in the genes *pck* and *odx* loci, the P1 promoter of the TCA cycle’s gene *gltA* was deleted, and the nature promoter of glutamate dehydrogenase-coding gene *gdh* was replaced by P_tac-M_ promoter that resulted in the final engineered strain *C. glutamicum* JL-69P_tac-M_ *gdh*. Furthermore, the suitable addition of biotin accelerates the l-lysine production in strain JL-69P_tac-M_ *gdh* because it elastically adjusts the carbon flux for cell growth and precursor supply. The final strain JL-69P_tac-M_ *gdh* could produce 181.5 ± 11.74 g L^−1^ of l-lysine with a productivity of 3.78 g L^−1^ h^−1^ and maximal specific production rate (*q*_Lys, max._) of 0.73 ± 0.16 g g^−1^ h^−1^ in fed-batch culture during adding 2.4 mg L^−1^ biotin with four times.

**Conclusions:**

Our results reveal that sufficient biomass, OAA and l-glutamate are equally important in the development of l-lysine high-yielding strain, and it is the first time to verify that fed-batch biotin plays a positive role in improving l-lysine production.

**Electronic supplementary material:**

The online version of this article (10.1186/s12934-018-0958-z) contains supplementary material, which is available to authorized users.

## Background

l-Lysine, one of the eight essential amino acids for animals and humans, has been applied in more and more fields, such as feed additives, dietary supplements as well as ingredient of pharmaceuticals and cosmetics [1]. With the widespread use and the increasing consumption of l-lysine, the strains with excellent productive performances and the perfect producing process are needed for fermentation to reduce production cost. Currently, the industrial l-lysine producers are almost *Corynebacterium glutamicum* or its subspecies, which have been created by multiple random mutagenesis and selections or by systems metabolic engineering [2, 3]. However, the strains created by mutation breeding exhibit many disadvantages, such as slow-growing, low sugar consumption rating, low stress tolerance [4, 5], systems metabolic engineering seems to be a “life-saving straw” for improving productive performances of l-lysine producers.

**Fig. 1:**
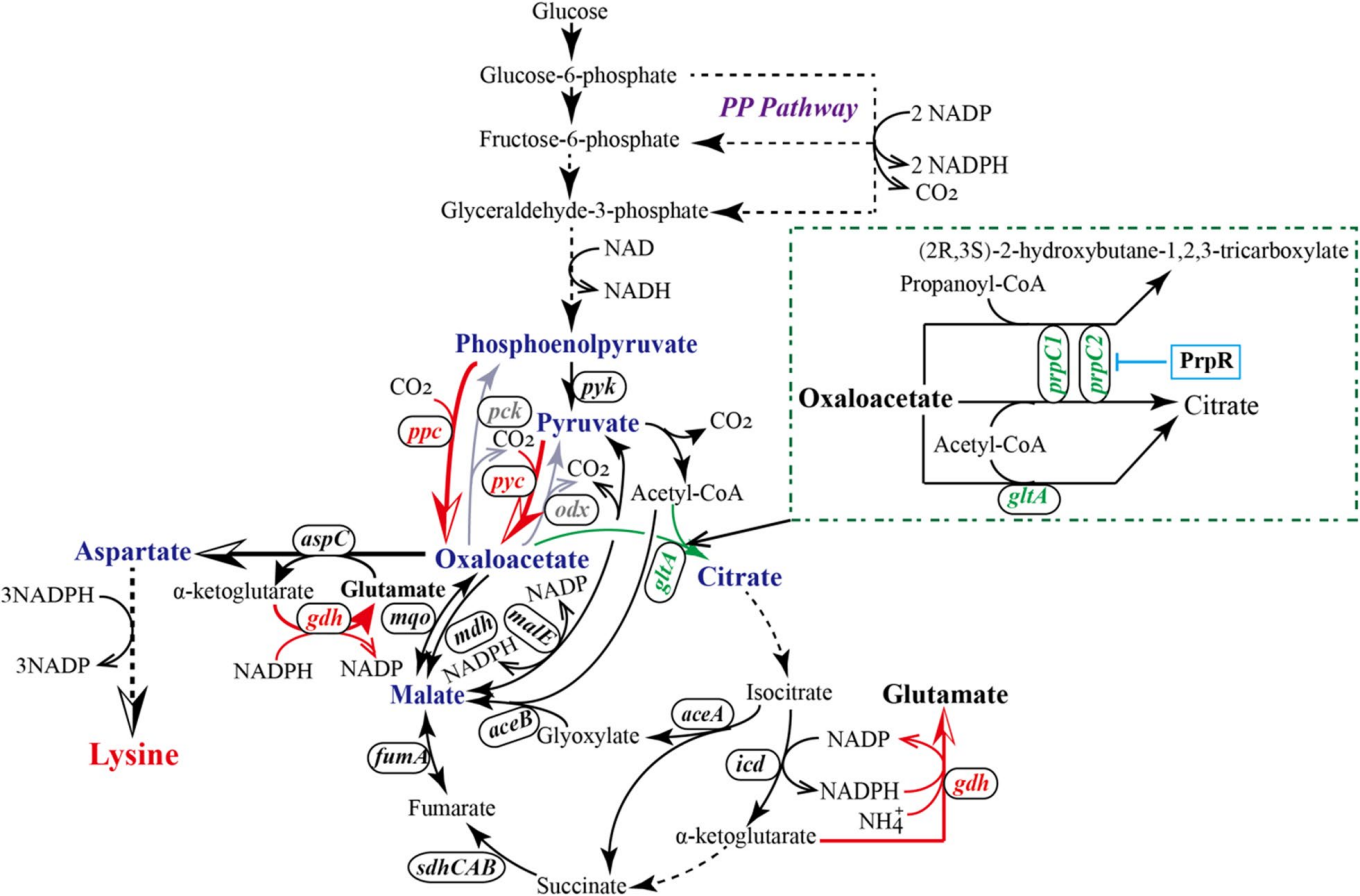
The central metabolic pathways of l-lysine in *C. glutamicum* (brief) and metabolic engineering strategy for constructing l-lysine high-yielding strain. Red arrows indicate amplification reactions; gray arrows indicate deletion reaction; green arrow indicates attenuation reaction. Italics indicate coding genes; dashed box indicates the reactions catalyzed by 2-methylcitrate synthases

As mentioned above, the biotin auxotrophic and non-pathogenic soil bacterium *C. glutamicum* has been widely applied in the fermentative production of l-lysine. At present, a various genes involved in l-lysine production were characterized at the molecular level, and subsequently, the l-lysine producers were achieved by genetic engineering of l-lysine biosynthetic pathway, central metabolic pathways as well as sugar uptake system in *C. glutamicum* [2, 4, 6–8]. One of the most prominent pathways in central metabolic pathways is the tricarboxylic acid (TCA) cycle, which provides several metabolic precursors and cofactors for cell growth and amino acids production [9]. As from Fig. 1, various factors play a part in regulating the carbon flux in TCA cycle, such as phosphoenolpyruvate (PEP)-pyruvate-oxaloacetate (OAA) node, glyoxysome, the biosynthetic pathway of l-lysine and l-glutamate, and the activities of pyruvate dehydrogenase complex as well as citrate synthase (CS). OAA, as a most important precursor for l-lysine, is a key component in PEP–pyruvate–OAA node, thus modifying PEP–pyruvate–OAA node is considered an important target for improving l-lysine production. However, OAA is also a key intermediate in TCA cycle, which provides metabolites and energy for cell growth, and for amino acid biosynthesis [10]. As the first critical enzyme, CS (encoded by *gltA* gene) catalyzes the polymerization of OAA with acetyl-CoA to form citrate, indicating that reducing the activity of CS will enhance l-lysine production because of the increased OAA supply [8]. However, the change of CS activity affects the cell growth [11, 12]. Therefore, properly adjusting CS activity to balance the cell growth and precursor supply is the wisest choice to increase the l-lysine yield and productivity.

l-Glutamate is another amino acid using an intermediate in TCA cycle as precursor, which is synthesized by reductive amination reaction of α-ketoglutarate (α-KG; Fig. 1), and this reaction is catalyzed by glutamate dehydrogenase (GDH, encoded by *gdh* gene) [13]. More importantly, l-glutamate is used as amino donor for l-lysine biosynthesis, which participates in the amination of OAA to form l-aspartate and the amination of *N*-succinyl-2-amino-6-ketopimetate to form *N*-succinyl-2,6-l,l-diaminopimelate [5]. In theory, improving the availability of l-glutamate should make the increase of l-lysine production. Strange that we rarely think of l-glutamate as parameter we should investigate. Under normal circumstances, l-glutamate is enough to supply the amino for l-lysine biosynthesis, but it’s inevitably encountered some special circumstances, such as the strain with attenuation of TCA cycle [14]. Moreover, l-lysine biosynthesis is closely related to the biosynthesis of l-glutamate. For example, biotin has different effects on the biosynthesis of l-lysine and l-glutamate. Many researches indicated that l-glutamate accumulation in *C. glutamicum* is induced by adding sub-optimal amounts of biotin [15], whereas l-lysine production in *C. glutamicum* is positively impacted by biotin because of improving the activity of biotin-dependent pyruvate carboxylase [16]. Furthermore, addition of biotin enhanced cell growth of *C. glutamicum* in glucose minimal medium [17]. Therefore, how to make develop equally is a problem that researchers pay attention to provide the l-glutamate availability and maintain the appropriate cell growth, in which they can keep the increase in l-lysine production.

Given the importance of TCA cycle in supplying l-lysine precursors and in affecting the cell growth of *C. glutamicum*, the present study was focus on the development of an l-lysine high-yielding strain via rationally modify the carbon flux of TCA cycle. Firstly, PEP–pyruvate–OAA node was modified to improve OAA supply. Secondly, the activity of CS was precisely adjusted to better distribution of OAA in TCA cycle, either into l-lysine biosynthetic pathway or into TCA cycle. Thirdly, changing the GDH activity via replacing the different promoters was executed to investigate the effect of l-glutamate on l-lysine production. Finally, to make up the defects of cell growth, the biotin was added, and the effect of its additive amount and adding manner on cell growth and l-lysine production were also discussed. Fed-batch fermentation of the final strain, the l-lysine production reached to 181.5 ± 11.74 g L^−1^ with a productivity of 3.78 g L^−1^ h^−1^ and maximal specific production rate (*q*_Lys, max._) of 0.73 ± 0.16 g g^−1^ h^−1^. Our study provided, for the first time, the definite effects of l-glutamate on l-lysine production in *C. glutamicum* with damaged TCA cycle. These results demonstrate once again the sufficient biomass is a prerequisite for gaining the high yield of target products.

## Results and discussion

### Metabolic engineering PEP–pyruvate–OAA node to increase processor OAA supply

Previous reports indicated that PEP–pyruvate–OAA node play an important role in cell growth and metabolites production, because it interconnects four central metabolic pathways of carbon metabolism, such as glycolytic pathway, anaplerotic pathway, gluconeogenesis and TCA cycle [18, 19]. It has been verified that OAA is a most important precursor for l-lysine biosynthesis [5, 8]. Increasing the replenishment or/and decreasing the consumption of OAA is beneficial to improving the l-lysine production. In order to increase the availability of OAA, we genetically modified the key enzymes in PEP–pyruvate–OAA node from *C. glutamicum* JL-6.

As can be seen from Fig. 1, ten enzymes involved in OAA metabolism were detected in crude extracts of *C. glutamicum* JL-6 (Table 1 and Additional file 1: Table S3), such as PEP carboxylase (PEPCx), PEP carboxykinase (PEPCk), pyruvate carboxylase (PCx), OAA decarboxylase (ODx), CS, malate:quinone oxidoreductase (MQO), malate dehydrogenase (MDH), malic enzyme (MalE), aspartate aminotransferase (AAT) and pyruvate kinase (PK). The first four enzymes among them are the key enzymes in PEP–pyruvate–OAA node. PEPCk (encoded by *pck* gene) and ODx (encoded by *odx* gene) involve in the consumption of OAA, whereas PEPCx (encoded by *ppc* gene) and PCx (encoded by *pyc* gene) participate in the replenishment of OAA pool [20, 21]. Consistent with the previous results [19], inactivation of PEPCk or ODx did not significantly increase the l-lysine production under aerobic conditions (Additional file 1: Table S4). Furthermore, the cell growth and by-products accumulation had not significantly changed during inactivation of PEPCk or ODx (Additional file 1: Table S4). However, the strain with deficient activity of PEPCk and ODx increased the l-lysine production to some extent and, conversely, the accumulation of pyruvate-family amino acids (PFAAs; e.g., l-alanine and l-valine) was slightly decreased (Additional file 1: Table S4). These results indicated that the availability of OAA did not significantly increase by only blocking the OAA consumption.

**Table 1:** In vitro specific activities of enzymes in genetically modified *C. glutamicum* strains and original strain *C. glutamicum* JL-6 as well as wild-type *C. glutamicum* ATCC13032

*C. glutamicum* strains	Specific activity of (U mg^−1^ protein)				
	CS	MCS1 and/or MCS2		AAT	GDH
		Acetyl-CoA	Propionyl-CoA		
ATCC13032	2.17 ± 0.11	nd	0.04 ± 0.00	0.43 ± 0.02	1.96 ± 0.32
JL-6	1.61 ± 0.06	nd	0.07 ± 0.03	0.66 ± 0.07	1.10 ± 0.17
JL-68	1.92 ± 0.02	nd	0.06 ± 0.02	0.70 ± 0.04	1.16 ± 0.14
JL-68∆*gltA*	0.05 ± 0.00	0.06 ± 0.01	0.05 ± 0.00	0.27 ± 0.03	0.65 ± 0.11
JL-68∆*ramA*	0.22 ± 0.03	0.04 ± 0.00	0.05 ± 0.01	0.78 ± 0.07	1.24 ± 0.17
JL-68∆P1*gltA* (or JL-69)	0.31 ± 0.02	0.05 ± 0.00	0.04 ± 0.02	0.83 ± 0.05	1.31 ± 0.21
JL-68∆P12*gltA*	0.05 ± 0.00	0.07 ± 0.01	0.05 ± 0.00	0.25 ± 0.08	0.57 ± 0.06
JL-68P_dapA-L1_ *gltA*	0.13 ± 0.01	0.09 ± 0.04	0.06 ± 0.02	0.73 ± 0.04	1.14 ± 0.07
JL-68∆*prpC1*	1.89 ± 0.06	< 0.0	< 0.0	0.69 ± 0.05	1.16 ± 0.13
JL-68 ∆*prpC2*	1.91 ± 0.09	< 0.01	< 0.01	0.69 ± 0.09	1.19 ± 0.16
JL-68∆*prpC1*∆*prpC2*	1.88 ± 0.03	–	–	0.68 ± 0.06	1.15 ± 0.11
JL-68∆*gltA*∆*prpC1*	< 0.01	–	–	nd	nd
JL-68∆*gltA*∆*prpC2*	≤ 0.01	–	–	nd	nd
JL-68∆*gltA*∆*prpC1*∆*prpC2*	ng	nd	nd	nd	nd
JL-68∆*gltA*∆*prpC1prpR*^G977A^	0.21 ± 0.02	0.32 ± 0.13	0.33 ± 0.15	0.79 ± 0.01	1.27 ± 0.17
JL-69P_dapA-L1_ *gdh*	0.21 ± 0.03	0.04 ± 0.01	0.31 ± 0.08	0.23 ± 0.07	0.21 ± 0.04
JL-69P_tac_ *gdh*	0.24 ± 0.02	0.05 ± 0.00	0.31 ± 0.11	0.92 ± 0.10	1.74 ± 0.17
JL-69P_tac-M_ *gdh*	0.27 ± 0.05	0.05 ± 0.01	0.32 ± 0.09	1.00 ± 0.07	2.56 ± 0.24
JL-69P_tuf_ *gdh*	0.31 ± 0.02	0.07 ± 0.03	0.34 ± 0.14	1.11 ± 0.21	3.98 ± 0.32
JL-69P_sod_ *gdh*	0.30 ± 0.01	0.06 ± 0.00	0.33 ± 0.06	1.04 ± 0.18	3.07 ± 0.15

All data are meaning values of three determinations of three independent experiments with ± SD

*CS* citrate synthase, *MCS* methylcitrate synthases, *AAT* aspartate aminotransferase, *GDH* glutamate dehydrogenase, *ng* not growth on medium used, *nd* not done, *–* not detected

**Fig. 2:**
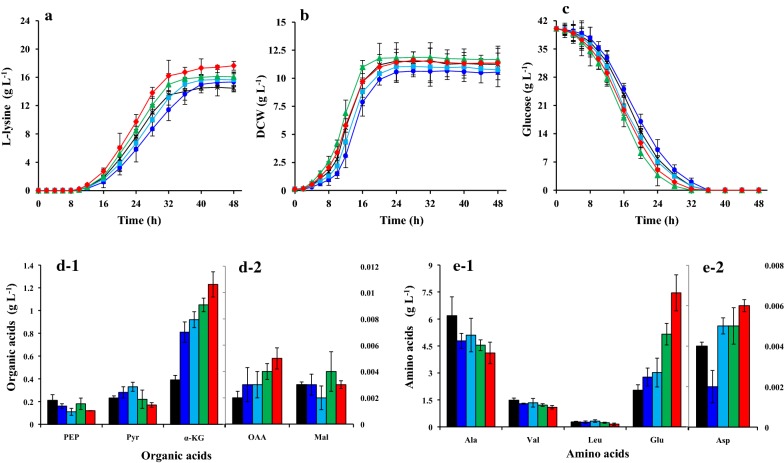
Comparison of l-lysine production (**a**), cell growth (**b**), glucose consumption (**c**), by-products accumulation [organic acids (**d**) and amino acids (**e**)] of *ppc* or/and *pyc* overexpression strains in shake-flasks culture with CgXIIM minimal medium containing 40 g L^−1^ glucose. **d**-**1**, **e**-**1** The data refers the left ordinate values, whereas the data in **d**-**2** and **e**-**2** refers the right ordinate values. Signal denotes: JL-6 (times, the black lines or bar), JL-65 (circle, the blue lines or bars), JL-66 (square, the light blue lines or bars), JL-67 (triangle, the green lines or bars), and JL-68 (diamond, the red lines or bars). The data represent mean values and standard deviations obtained from three independent cultivations

Furthermore, the next modification aimed at increasing the replenishment of OAA pool was executed by increase the flux in anaplerotic pathway. In contrast to *E. coli* [22], *C. glutamicum* possesses two anaplerotic enzymes, i.e., PEPCx and PCx [21]. Although PCx is a major enzyme for OAA supply in *C. glutamicum* [23], both of them played a part in cell growth and amino acid production during growth on glucose [24]. For all this, we constructed *ppc*-overexpressing strain, *pyc*-overexpressing strain, and *ppc* and *pyc*-dual-overexpressing strain (Additional file 1: Fig. S1), and l-lysine, residual glucose concentration as well as cell growth were monitored over the cause of the experiment. The strains *C. glutamicum* JL-6 ∆*pck*::*ppc* (i.e., *C. glutamicum* JL-66), *C. glutamicum* JL-6 ∆*odx*::*pyc* (i.e., *C. glutamicum* JL-67), and *C. glutamicum* JL-6 ∆*pck*::*ppc* ∆*odx*::*pyc* (i.e., *C. glutamicum* JL-68) exhibited an increase in the corresponding enzyme activity, especially for PCx (Additional file 1: Table S3). This is because the activity of nature PCx is too low in *Corynebacterium* species [25]. Accordingly, overexpression of the anaplerotic enzymes resulted in a marked rise in l-lysine production as compared with *C. glutamicum* JL-6 or *C. glutamicum* JL-65 (Fig. 2a). As reported by Peters-Wendisch et al. [23], overexpression of PCx is better than overexpression of PEPCx for increasing l-lysine production (Fig. 2a). The *pyc*-overexpressing strain *C. glutamicum* JL-67 excreted 16.07 ± 0.41 g L^−1^ of l-lysine with a maximal specific production rate (*q*_Lys, max._) of 0.31 ± 0.05 g g^−1^ h^−1^, whereas the *ppc*-overexpressing strain *C. glutamicum* JL-66 only accumulated 15.65 ± 1.05 g L^−1^ of l-lysine with a *q*_Lys, max_ of 0.29 ± 0.04 g g^−1^ h^−1^. Interestingly, overexpression of the anaplerotic enzymes increased cell growth to some extent (Fig. 2b). The reason may be laid out in the report by Peters-Wendisch et al. [26], who found that PEPCx and PCx dual-deficient mutant was unable to grow on glucose as sole carbon sources. In order to meet the need of carbon sources, the consumption rate of glucose was improved during overexpression of anaplerotic enzymes (Fig. 2c). In addition, overexpression of PCx decreased the accumulation of PFAAs because of the decreased pyruvate concentration, whereas overexpression of PEPCx did the opposite (Fig. 2d, e). *C. glutamicum* possess the complicated PEP-pyruvate-OAA node [18], the intracellular OAA can be decarboxylated to form pyruvate by ODx, thus serving as a precursor for PFAAs biosynthesis [27]. It is natural that simultaneous overexpression of PEPCx and PCx remarkably increased l-lysine production (increased to 17.63 ± 0.61 g L^−1^), and decreased the accumulation of pyruvate and PFAAs (Fig. 2). The effects of malic enzyme, another enzyme in PEP-pyruvate-OAA node, on l-lysine production were also investigated in this study, but there is no obvious function during modification of malic enzyme (Additional file 1: Tables S3 and S4). Because of the positive effects of PEPCx and PCx on l-lysine production, we chose PEPCx and PCx dual-overexpression strain *C. glutamicum* JL-68 as target strain for follow-up study.

### Properly adjusting tricarboxylate synthase activities to increase processor OAA supply

The above results showed that although the accumulation of pyruvate and PFAAs was decreased, the availability of OAA for l-lysine production was not sufficient. In addition, the accumulation of by-products derived from TCA cycle (e.g., α-KG and l-glutamate) was increased during overexpression of anaplerotic enzymes (Fig. 2d, e). These results indicated that more OAA was pulled into TCA cycle rather than into AAT-catalyzed reaction [28]. Therefore, a precisely adjusted the carbon flux into TCA cycle can potentially increase l-lysine productivity. Our results and other researchers’ results showed that knockout of pyruvate dehydrogenase gene (i.e., ∆*aceE*) or mutation of isocitrate dehydrogenase gene (i.e., *icd*^A1G^) decreased the flux into TCA cycle, resulting in an increase of l-lysine production (Additional file 1: Fig. S2a) [1, 4, 5]. However, the pyruvate dehydrogenase-deficient strain was unable to grow on glucose as sole carbon sources unless supplemented with acetate (Additional file 1: Fig. S2b) [1].

CS (encoded by *gltA* gene) catalyzes the first step of TCA cycle (Fig. 1), and it is a rate-limiting enzyme for TCA cycle [29]. Shiio et al. [14] has proved that reducing CS activity leads to increase the availability of OAA. However, the activity of CS is too low to maintain the cell growth because TCA cycle supplies metabolites and energy for cell growth [8, 12]. Hence, subtle control of the CS activity is an effective strategy for increasing l-lysine productivity. In order to redirect carbon into TCA cycle in strain JL-68, a series of strain with different CS activity were constructed. In addition, we also compared the effects of different CS activity on cell growth, l-lysine productivity and by-products accumulation. Consistent with the previous results [8], deletion of the open reading frame of *gltA* gene (i.e., strain JL-68∆*gltA*) or its long promoter (i.e., strain JL-68∆P12*gltA*) greatly decreased the CS activity and hampered the cell growth (Tables 1, 2). Accordingly, these CS-deficient strains did not accumulate any l-lysine and by-products (Table 2; Additional file 1: Fig. S3). Whereas, deletion of the short promoter of *gltA* gene (i.e., strain JL-68∆P1*gltA*) still exhibited 0.31 ± 0.02 U (mg of protein)^−1^ of CS activity, which is 16.15% of activity of strain JL-68 (Table 1). Eikmanns et al. [30] have verified that the expression level of *C. glutamicum gltA* is weak during the lack of a typical ʻ− 35ʼ consensus sequence, and this might be one reason for the low CS activity in strain JL-68∆P1*gltA*. However, deletion of the long promoter P12 of gene *gltA* will stop the *gltA* gene expression [8]. Although the final DCW of strain JL-68∆P1*gltA* was decreased, the growth rate was the similar to the strain JL-68 (Table 2). More importantly, the l-lysine yield and *q*_Lys, max_ were significantly increased as compared with the strain JL-68 (Table 2). Furthermore, the accumulation of by-products (including organic acids except α-KG, OAA and malate, PFAAs and aromatic amino acids) were increased during deletion of the short promoter of *gltA* gene (Additional file 1: Fig. S3). This is because more carbon from OAA was pull into l-lysine biosynthetic pathway rather than into TCA cycle [8]. Following this thought, decrease of CS activity seems to be beneficial to improve l-lysine production. However, our results indicated that further reducing the CS activity did not further increase the l-lysine production (Table 2). For example, strain JL-68∆*ramA* with deletion of *ramA* gene (encoding RamA) only exhibited 11.46% of CS activity of the control train JL-68 (0.22 ± 0.03 vs. 1.92 ± 0.02 U mg^−1^ protein) (Table 1), whereas the l-lysine yield was only slightly higher than that of control strain JL-68, but lower than that of strain JL-68∆P1*gltA* (Table 2). More noticeably, although strain JL-68P_dapA-L1_ *gltA* with replacement of nature promoter by *dapA*-*L1* promoter showed specific activities of CS of 0.13 ± 0.01 U (mg of protein)^−1^, which is only 6.77% of the control train JL-68, the l-lysine yield was even lower than that of strain JL-68 (Table 2). Since RamA, one of transcriptional regulators of acetate metabolism, acts as an activator of *gltA* expression [31], thus knockout of RamA will lead to the decrease of CS activity. In addition, because P_dapA-L1_ is a weak promoter [32], the expression level of *gltA* gene is too low to exhibit a high CS activity during replacement of promoter. However, the CS activity was too low to provide enough carbon flux for TCA cycle in strains JL-68∆*ramA* and JL-68P_dapA-L1_ *gltA*, thus the needs (e.g., 4-carbon dicarboxylic acid and energy) for cell growth are not satisfied so that the cell growth was damaged (Table 2) [8, 11, 12]. Accordingly, the l-lysine yield was sharply decreased because of the excessively low biomass (Table 2).

**Table 2:** The DCW (24 h or 48 h), l-lysine production (24 h or 48 h), maximal specific growth rate (μ_max._), maximal specific production rate of l-lysine (*q*_Lys, max._) and glucose conversion efficiency (α) of *C. glutamicum* strains under the different of CS activity

*C. glutamicum* strains	CS (%)^a^	DCW (g L^−1^)		l-Lys yield (g L^−1^)		μ_max_^b^ (h^−1^)	*q*_Lys, max._^c^ (g g^−1^ h^−1^)	α (%)^d^
		24 h	48 h	24 h	48 h			
JL-68	100	11.48 ± 1.76	11.35 ± 1.52	10.60 ± 1.06	17.63 ± 0.61	0.21	0.34 ± 0.05	44.08
JL-68∆*gltA*	2.60	0.63 ± 0.00	0.96 ± 0.15	–	–	nd	nd	nd
JL-68∆*ramA*	11.46	6.03 ± 1.18	6.82 ± 2.05	8.01 ± 1.53	19.17 ± 3.45	0.15	0.53 ± 0.12	47.93
JL-68∆P1*gltA* (or JL-69)	16.15	9.67 ± 2.13	9.75 ± 1.35	9.54 ± 2.14	21.44 ± 1.93	0.20	0.49 ± 0.08	53.60
JL-68∆P12*gltA*	2.60	0.67 ± 0.05	1.01 ± 0.13	–	–	nd	nd	nd
JL-68P_dapA-L1_ *gltA*	6.77	2.23 ± 0.36	4.92 ± 0.81	5.01 ± 1.15	14.75 ± 2.16	0.11	0.41 ± 0.17	36.88
JL-68∆*prpC1*	98.44	11.24 ± 1.02	11.53 ± 1.17	10.58 ± 1.37	17.79 ± 1.23	0.22	0.38 ± 0.11	44.48
JL-68 ∆*prpC2*	99.48	11.29 ± 1.31	11.51 ± 1.13	10.56 ± 1.04	17.73 ± 1.05	0.22	0.37 ± 0.07	44.33
JL-68∆*prpC1*∆*prpC2*	97.92	11.25 ± 1.25	11.50 ± 0.87	10.57 ± 1.12	17.71 ± 1.12	0.22	0.37 ± 0.06	44.28
JL-68∆*gltA*∆*prpC1*	nd	0.14 ± 0.01	0.12 ± 0.02	–	–	nd	nd	nd
JL-68∆*gltA*∆*prpC2*	nd	0.15 ± 0.01	0.16 ± 0.01	–	–	nd	nd	nd
JL-68∆*gltA*∆*prpC1*∆*prpC2*	nd	0.13 ± 0.00	0.13 ± 0.01	–	–	nd	nd	nd
JL-68∆*gltA*∆*prpC1prpR*^G977A^	10.94	5.17 ± 1.01	6.03 ± 1.12	7.31 ± 1.15	20.18 ± 3.61	0.14	0.58 ± 0.33	50.45

All data are meaning values of three determinations of three independent experiments with ± SD

*nd* not done, *–* not detected

^a^The percent of CS activity in different genetically modified *C. glutamicum* strains and taken the CS activity in parental strain JL-68 as reference standard

^b^The maximal specific growth rate of cell in the whole fermentation cycle

^c^The maximal specific production rate of l-lysine in the whole fermentation cycle

^d^The conversion rate of glucose into l-lysine after cultivating 48 h

Deletion of RamA or replacement of *gltA* promoter has been shown to be ineffective in improving l-lysine production, because CS activity was too low (Tables 1, 2). Thus, we can infer that properly adjusting CS activity is a key requirement for increasing the l-lysine production. Many researches have pointed out that *C. glutamicum* possesses three tricarboxylate synthases: CS and two 2-methylcitrate synthases [MCS; i.e., MCS1 (encoded by *prpC1*) and MCS2 (encoded by *prpC2*)] [12]. Interestingly, MCS1 and MCS2 exhibit CS activity, which can catalyze the condensation reaction of OAA with both acetyl-CoA and propionyl-CoA to form citrate (Fig. 1) [33]. Moreover, the expression of *prpC1* and *prpC2* was regulated by the promoter PrpR [33], and Radmacher and Eggeling [12] has found that mutation of PrpR stimulates the expression of *prpC2*. In order to properly adjust and clear-cut the MCS flux, we construct a serial of MCS-deficient or PrpR-mutated strains with different MCS activity, and assay the effects of MCS on CS activity, cell growth and l-lysine production. Predictably, the original strains ATCC13032, JL-6 and JL-68 exhibited the low MCS activity with neither acetyl-CoA nor propionyl-CoA as substrate (Table 1), because the expression of *prpC1* and *prpC2* was induced by propionate but was inhibited by glucose [12]. Consistent with the previous results [12], the single or double deletion of *prpC1* and *prpC2* did not markedly influence CS activity, whereas observably decreased the MCS activity. However, in combined with the deletion of *gltA*, the CS activity was barely detectable. Interestingly, when *prpR* gene was mutated in strain JL-68∆*gltA*∆*prpC1*, resulting strain JL-68∆*gltA*∆*prpC1prpR*^G977A^, both CS and MCS activities were increased as compared with the strains JL-68∆*gltA* and JL-68∆*gltA*∆*prpC1* (Table 1). Growth and l-lysine production of these mutants were analyzed on the CgXIIM-medium with 40 g L^−1^ glucose (Table 2). Growth of strain JL-68∆*prpC1*, JL-68∆*prpC2* or JL-68∆*prpC1*∆*prpC2* was indistinguishable from that of strain JL-68, illustrating that two MCS are unessential for glucose metabolism and confirming previous results obtained with disruption of MCS-coding genes in l-lysine producer DM1800 [12]. But in the case of *gltA* disruption mutant, the strain grew poorly because CS is essential for glucose metabolism in *C. glutamicum* [30]. However, the growth of strain JL-68∆*gltA*∆*prpC1prpR*^G977A^ reached to 5.17 ± 1.01 g L^−1^ at 24 h and 6.03 ± 1.12 g L^−1^ at 48 h with a μ_max_ of 0.14 h^−1^ (Table 2). Moreover, the l-lysine production was recovered and reached to 20.18 ± 3.61 g L^−1^ with a *q*_Lys, max_ of 0.58 ± 0.33 g g^−1^ h^−1^ at 48 h (Table 2). This is because mutation of PrpR makes the strain with the ability of condensation of OAA and acetyl-CoA to form citrate [12]. Although the l-lysine production was increased to some extent during mutation of PrpR, there was lower than that of strain JL-68∆P1*gltA* (Table 2). This is because a proper level of CS is essential for cell growth (Table 2). It should be noted that the accumulation of by-products would be changed with the change of CS activity (Additional file 1: Fig. S3). For example, the accumulation of by-products derived from TCA intermediates (e.g., α-KG and l-glutamate) in strain JL-68∆P1*gltA* was significantly decreased as compared with the strain JL-68. Therefore, we chose P1-deficient strain *C. glutamicum* JL-68∆P1*gltA* as target strain for follow-up study, and designated as *C. glutamicum* JL-69.

### Modestly raising glutamate dehydrogenase activity to increase processor l-glutamate supply

As compared with the strain JL-68, the accumulation of l-glutamate was drastically decreased (from 7.45 ± 1.01 to 1.08 ± 0.13 g L^−1^), whereas the OAA concentration was markedly increased in strain JL-69 (from < 0.01 to 1.13 ± 0.14 g L^−1^; Fig. 2d, e and Table 3). These results indicated that OAA is not a limiting factor for l-lysine production in strain JL-69. However, it is generally known that l-glutamate is used as amino donor for l-lysine biosynthesis [5, 14]. Therefore, the availability of l-glutamate may limit the l-lysine production in strain JL-69. In order to investigate the effect of l-glutamate on l-lysine production, we modified the GDH activity by replacing the promoter of GDH-coding gene *gdh* in strain JL-69. Five classes of promoters (i.e., P_dapA-L1_, P_tac_, P_tac-M_, P_tuf_ and P_sod_) were used in this study.

**Table 3:** The DCW, l-lysine production, by-product accumulation, maximal specific growth rate (μ_max._), maximal specific production rate of l-lysine (*q*_Lys, max._) and glucose conversion efficiency (α) of modified glutamate dehydrogenase *C. glutamicum* strains

*C. glutamicum* strains	Concentration of (g L^−1^)					DCW (g L^−1^)	μ_max_ (h^−1^)	*q*_Lys, max._ (g g^−1^ h^−1^)	α (%)
	l-Lys	l-Asp	l-Glu	α-KG	OAA				
JL-69	21.44 ± 1.93	< 0.01	1.08 ± 0.13	0.77 ± 0.21	1.13 ± 0.14	9.75 ± 1.35	0.20	0.47 ± 0.08	53.60
JL-69P_dapA-L1_ *gdh*	10.75 ± 1.14	< 0.01	0.22 ± 0.06	1.38 ± 0.12	1.22 ± 0.17	9.82 ± 1.01	0.22	0.21 ± 0.03	26.88
JL-69P_tac_ *gdh*	22.27 ± 1.29	< 0.01	2.05 ± 0.18	0.65 ± 0.13	0.96 ± 0.08	9.24 ± 1.35	0.20	0.48 ± 0.08	55.68
JL-69P_tac-M_ *gdh*	23.86 ± 2.16	0.21 ± 0.03	3.38 ± 0.42	0.41 ± 0.10	0.32 ± 0.05	7.83 ± 1.12	0.19	0.63 ± 0.11	59.65
JL-69P_tuf_ *gdh*	16.03 ± 1.32	< 0.01	1.82 ± 0.07	0.18 ± 0.03	< 0.01	5.36 ± 1.04	0.14	0.81 ± 0.17	40.08
JL-69P_sod_ *gdh*	20.16 ± 2.08	< 0.01	1.27 ± 0.10	0.24 ± 0.09	< 0.01	6.78 ± 0.72	0.16	0.70 ± 0.08	50.40

All data are meaning values of three determinations of three independent experiments with ± SD

*l-Lys* l-lysine, l*-Asp* l-aspartate, l*-Glu* l-glutamate; *α-KG* α-ketoglutarate, *OAA* oxaloacetate

As shown in Table 1, the GDH activity was changed along with replacing the different promoters. Since the P_dapA-L1_ is a weak promoter [32], the GDH activity in strain JL-69P_dapA-L1_ *gdh* was only 0.21 ± 0.04 U (mg protein)^−1^, which was about 16.03% of the activity in strain JL-69. Nevertheless, the GDH activity was significantly increased during replacing the native promoter with P_tuf_ and P_sod_ (increased by 203.82 and 134.35%, respectively) because the P_tuf_ and P_sod_ are the strong promoters in *C. glutamicum* [4]. In addition, Xu et al. [34] pointed out that the P_tac_ is a moderate promoter in *C. glutamicum*, thus the strain JL-69P_tac_ *gdh* had a moderate increase of GDH activity, reached to 1.74 ± 0.17 U (mg protein)^−1^ (Table 1). However, replacing the − 10 region of the P_tac_ with the sequence of “TGTGGTACCATGT”, designated as promoter P_tac-M_, increased the GDH-coding gene *gdh* expression level, thus further increased the GDH activity (Table 1), which is consistent with the previous results reported by Xu et al. [34]. Consistent with the results reported by Asakura et al. [35], the l-glutamate concentration was increased during increase of GDH activity (Table 3).

Cell growth and l-lysine production of strains with different GDH activity were analyzed on the CgXIIM-medium with 40 g L^−1^ glucose (Table 3). Strains JL-69, JL-69P_dapA-L1_ *gdh* and JL-69P_tac_ *gdh* had nearly equal DCW and growth rate, i.e., 9.75 ± 1.35 g L^−1^ (μ_max_ = 0.20 h^−1^), 9.82 ± 1.01 g L^−1^ (μ_max_ = 0.22 h^−1^) and 9.24 ± 1.35 g L^−1^ (μ_max_ = 0.20 h^−1^), respectively. However, the DCW and growth rate were decreased along with the further increase of GDH activity, especially for strains JL-69P_tuf_ *gdh* and JL-69P_sod_ *gdh* (Table 3). The DCW of strains JL-69P_tuf_ *gdh* and JL-69P_sod_ *gdh* was 5.36 ± 1.04 g L^−1^ with a μ_max_ of 0.14 h^−1^ and 6.78 ± 0.72 g L^−1^ with a μ_max_ of 0.14 h^−1^, respectively. This is because more α-KG in TCA cycle was pulled into l-glutamate biosynthetic pathway, and thus led to decrease the carbon and energy for cell growth [36]. This conclusion has been confirmed in analyzing the accumulations of l-lysine and by-products in fermented broth (Table 3 and Additional file 1: Fig. S4). The highest increase of l-lysine production (reached to 23.86 ± 2.16 g L^−1^) was observed for strain JL-69P_tac-M_ *gdh*, but the increase was slightly higher for strain JL-69P_tac_ *gdh* (reached to 22.27 ± 1.29 g L^−1^). However, the l-lysine production was drastically decreased during replacing the native promoter of *gdh* gene with weak promoter P_dapA-L1_ (Table 3), whereas the accumulations of by-products (such as PEP, pyruvate, lactate, OAA, α-KG, PFAAs, etc.) were increased to some extent (Additional file 1: Fig. S4). This is because l-glutamate is in short supply (Table 3). Interestingly, the l-lysine production was also decreased during replacing with the strong promoters P_tuf_ and P_sod_, but it is mostly due to a limited biomass rather than l-glutamate. However, the maximal specific production rate of l-lysine (*q*_Lys, max._) was observably increased in strain JL-69P_tuf_ *gdh* and JL-69P_sod_ *gdh*, which was higher than that of strain JL-69P_tac-M_ *gdh* (Table 3). In addition, in order to eliminate the coupling effects of CS and GDH, we investigated the cell growth and l-lysine production in strain JL-68∆*gltA*∆*prpC1prpR*^G977A^ (designated as *C. glutamicum* JL-610) with different GDH activity. Unlike the changes in strain JL-69, replacement of native *gdh* promoter with promoter P_tac_ is beneficial for l-lysine production in strain JL-610, which is better than that of replacement with promoter P_tac-M_ (Additional file 1: Table S5). However, the l-lysine yield in strain JL-610P_tac_ *gdh* was lower than that of in strain JL-69P_tac-M_ *gdh* (Table 3 and Additional file 1: Table S5). Therefore, we chose strain *C. glutamicum* JL-69P_tac-M_ *gdh* as target strain for follow-up study.

### Gradually adding biotin to maintain the relatively constant biomass in culture

According to the above results, the sufficient biomass in the fermentation system is crucial to increase the l-lysine yield and productivity (Table 3 and Additional file 1: Table S5). As a growth-stimulating factor, biotin acts as coenzyme for many enzymes, which involve in gluconeogenesis, glyoxysome, fatty acid biosynthesis, etc. [17]. Many researches indicated that the proper amount of biotin enhances cell growth and the production of the target products [14, 37]. Consistent with the previous results [14], the cell growth was increased with increasing the biotin addition for strains JL-69P_tac-M_ *gdh*, JL-69P_tuf_ *gdh* and JL-69P_sod_ *gdh* in shake-flask culture (Table 4). However, the increased l-lysine production appeared in the condition of no more than 0.6 mg L^−1^ biotin for strain JL-69P_tac-M_ *gdh*, but no more than 0.8 mg L^−1^ for strains JL-69P_tuf_ *gdh* and JL-69P_sod_ *gdh*. Conversely, the l-lysine production would decrease (Table 4). The same phenomenon was observed by Shiio et al. [14], and it is because target products are completely oxidized into CO_2_ and to form other products, for example acetate [38]. The strain JL-69P_tac-M_ *gdh* accumulated 25.24 ± 1.74 g L^−1^ of l-lysine during addition of 0.6 mg L^−1^ biotin. In order to better understand the mechanism of added biotin, we investigated the relative expression level of the genes involved in TCA cycle of strain JL-69P_tac-M_ *gdh* between addition of 0.2 and of 0.6 mg L^−1^ biotin. The relative expression level of most the tested genes (including *pyc*, *aceE*, *malE*, *gltA*, *aceA*, *aceB*, *sdhA*, *sdhB*, *fumA*, *mqo* and *aspC* genes) in 0.6 mg L^−1^ biotin-culture were higher than that of in 0.2 mg L^−1^ biotin-culture except the *ppc*, *icd*, *mdh* and *gdh* genes (Additional file 1: Fig. S5). Pejin and Razmovski [39] proved that addition of biotin promotes the regeneration of oxaloacetate because of the increased activity of PCx and isocitrate lyase (encoded by gene *aceA*). Isocitrate lyase is a key enzyme in glyoxysome, thus the expression level of genes in glyoxysome will be increased during addition of biotin. When adding 0.8 mg L^−1^ biotin, the l-lysine production in strains JL-69P_tuf_ *gdh* and JL-69P_sod_ *gdh* reached to 22.47 ± 1.32 and 24.21 ± 1.05 g L^−1^ respectively, which were lower than that of strain JL-69P_tac-M_ *gdh* (25.24 ± 1.74 g L^−1^) during addition of 0.6 mg L^−1^ biotin (Table 4). These results reconfirmed that strain JL-69P_tac-M_ *gdh* is better than strains JL-69P_tuf_ *gdh* and JL-69P_sod_ *gdh* for l-lysine production.

**Table 4:** The DCW, l-lysine production, maximal specific growth rate (μ_max._), and maximal specific production rate of l-lysine (*q*_Lys, max._) of genetically defined *C. glutamicum* strains in the shaking culture and under the different concentration of biotin

Biotin conc. (mg L^−1^)	JL-69P_tac-M_ *gdh*				JL-69P_tuf_ *gdh*				JL-69P_sod_ *gdh*			
	Lys conc. (g L^−1^)	DCW (g L^−1^)	μ_max_ (h^−1^)	*q*_Lys, max._(g g^−1^ h^−1^)	Lys conc. (g L^−1^)	DCW (g L^−1^)	μ_max_ (h^−1^)	*q*_Lys, max_ (g g^−1^ h^−1^)	Lys conc. (g L^−1^)	DCW (g L^−1^)	μ_max_ (h^−1^)	*q*_Lys, max._ (g g^−1^ h^−1^)
0.2	23.86 ± 2.16	7.53 ± 1.12	0.17	0.63 ± 0.11	16.03 ± 1.32	5.36 ± 1.04	0.14	0.81 ± 0.17	20.16 ± 2.08	6.78 ± 0.72	0.16	0.70 ± 0.08
0.3	23.93 ± 1.28	8.72 ± 1.04	0.19	0.61 ± 0.06	17.10 ± 1.23	7.71 ± 1.36	0.18	0.64 ± 0.04	20.63 ± 3.15	8.03 ± 1.02	0.19	0.67 ± 0.13
0.4	24.36 ± 2.11	9.25 ± 1.13	0.23	0.63 ± 0.14	19.24 ± 1.48	8.82 ± 1.44	0.21	0.65 ± 0.12	21.32 ± 1.77	9.10 ± 1.21	0.22	0.67 ± 0.05
0.5	25.05 ± 2.16	9.76 ± 1.09	0.23	0.69 ± 0.13	20.21 ± 1.12	9.38 ± 0.95	0.22	0.63 ± 0.20	22.44 ± 2.13	10.07 ± 0.58	0.26	0.65 ± 0.14
0.6	25.24 ± 1.74	10.23 ± 2.04	0.25	0.68 ± 0.11	21.26 ± 2.05	10.16 ± 1.01	0.25	0.66 ± 0.13	24.05 ± 1.66	10.38 ± 0.42	0.26	0.66 ± 0.03
0.8	24.47 ± 2.18	11.35 ± 1.18	0.28	0.62 ± 0.16	22.47 ± 1.32	10.83 ± 2.11	0.29	0.67 ± 0.07	24.21 ± 1.05	11.11 ± 1.03	0.31	0.63 ± 0.09
1.0	21.83 ± 2.10	13.14 ± 1.05	0.33	0.56 ± 0.05	20.82 ± 1.74	12.96 ± 1.02	0.33	0.59 ± 0.04	22.15 ± 2.12	12.76 ± 1.15	0.32	0.62 ± 0.11
1.2	18.29 ± 1.93	16.59 ± 2.06	0.39	0.43 ± 0.08	19.13 ± 1.02	15.38 ± 1.17	0.34	0.50 ± 0.12	21.02 ± 1.76	15.49 ± 1.26	0.35	0.55 ± 0.06

**Fig. 3:**
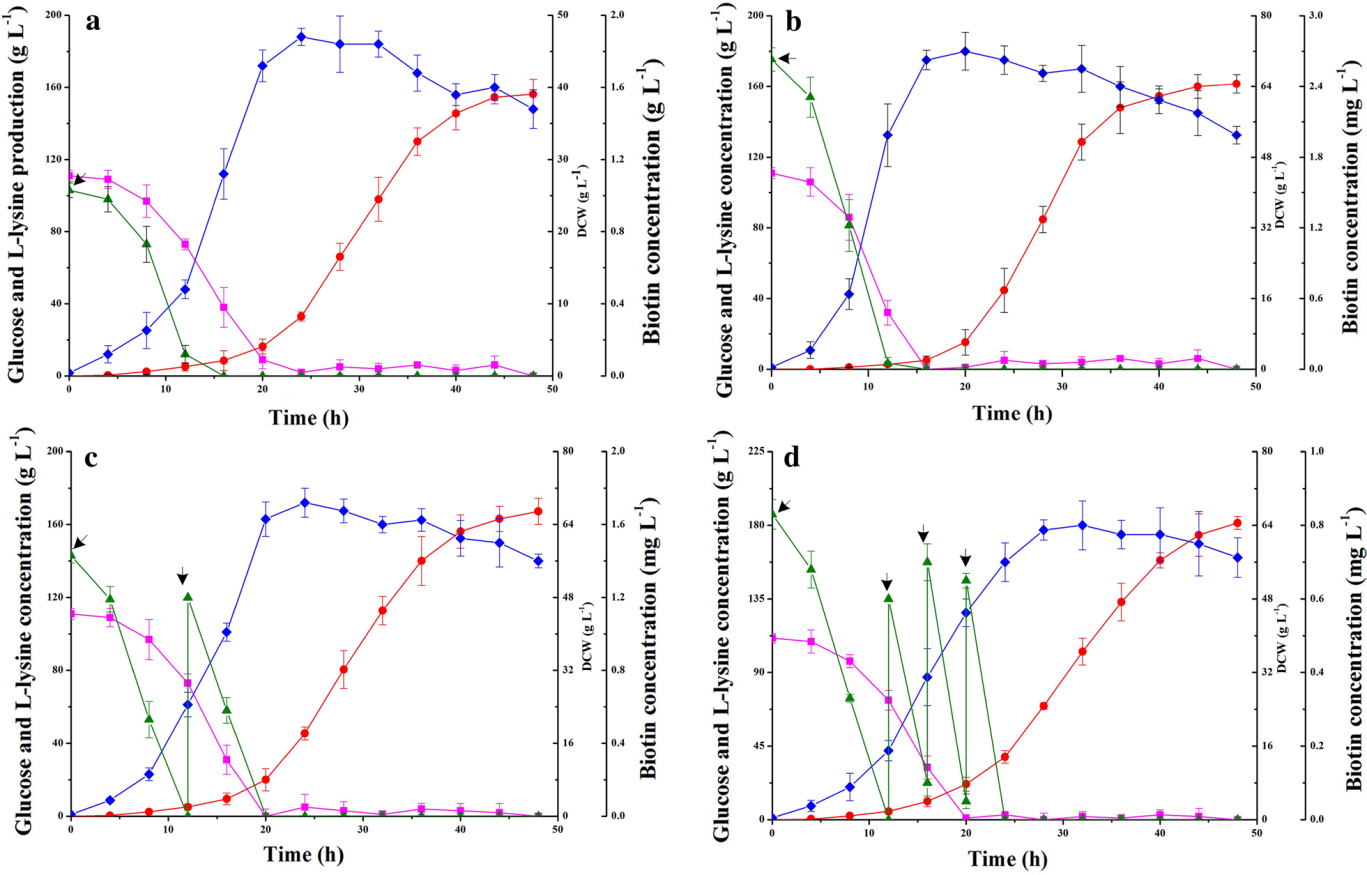
The effect of additive amount and adding manner of biotin on cell growth, glucose consumption and l-lysine production of strain JL-69P_tac-M_ *gdh* in fed-batch fermentation. Right pointing arrow represents the point-in-time of biotin adding. **a** 0.8 mg L^−1^ of biotin was added in each time; **b** 2.4 mg L^−1^ of biotin was added in each time; **c** 1.2 mg L^−1^ of biotin was added in each time; **d** 0.6 mg L^−1^ of biotin was added in each time. Signal denotes: DCW (filled diamond, blue line), glucose (filled square, magenta line), l-lysine (filled circle, red line), and biotin (filled triangle, olive line). The data represent mean values and standard deviations obtained from three independent cultivations

In addition, fed-batch biotin was carried out in a 5-L jar fermenter containing 1.0 L fermentation media to test the production performance of strain JL-69P_tac-M_ *gdh* under different concentration of biotin. Compared with adding 0.8 mg L^−1^ biotin, adding 2.4 mg L^−1^ biotin had the trend to increase the l-lysine production (Fig. 3a, b). Moreover, the different mode of biotin feeding can also affect the l-lysine production in strain JL-69P_tac-M_ *gdh*. When adding times reach four (0.6 mg L^−1^/time), the l-lysine production increased to 181.5 ± 7.65 g L^−1^, which was higher than that of added once (accumulated 161.5 ± 9.04 g L^−1^ of l-lysine) or twice (1.2 mg L^−1^/time; accumulated 167.2 ± 5.87 g L^−1^ of l-lysine) (Fig. 3b–d). This may be that more carbon flux was used to synthesize biomass during the post-exponential phase, but most of cells were cracked at the end of fermentation (Fig. 3). The excellent performance of strain JL-69P_tac-M_ *gdh* is also seen in the glucose conversion efficiency (α) and productivity during adding biotin with four times (reached to 64.6% and 3.78 g L^−1^ h^−1^, respectively). Although the productivity of strain JL-69P_tac-M_ *gdh* is lower than that of strain LYS-12 reported by Becker et al. [4], the final titer is higher than that one (181.5 g L^−1^ vs. 120 g L^−1^). Thus, the strain JL-69P_tac-M_ *gdh* has great potential to produce l-lysine in industrial-scale with the strategy of fed-batch biotin.

## Conclusions

In this work, we have studied on the development of a high-yielding strain for the production of l-lysine by reasonable adjusting precursors supply (including OAA and l-glutamate), and confirm the importance of biotin and its feeding mode for increasing the l-lysine production. Many researches pointed out that TCA cycle provides precursors (including OAA and l-glutamate) for cell growth and l-lysine production [4, 40, 41], so reasonable adjusting the carbon flux of TCA cycle is beneficial to balance cell growth and precursors supply which in turn will increase the l-lysine production. As shown, the engineered strain JL-69P_tac-M_ *gdh* excreted 156.3 ± 5.16 g L^−1^ of l-lysine with α of 59.7% and productivity of 3.26 g L^−1^ h^−1^. Furthermore, fed-batch biotin plays a positive role in improving l-lysine production. Adding biotin with four times, the l-lysine production reached to 181.5 ± 7.65 g L^−1^, and the α and productivity are 64.6% and 3.78 g L^−1^ h^−1^, respectively. Compared with the previous results [4], however, the productivity strain JL-69P_tac-M_ *gdh* of is low. Therefore, further improving l-lysine production with strain JL-69P_tac-M_ *gdh* will aim at shortening the fermentation time by enhancing substrate uptake rate (including carbon source and nitrogen source) and further optimizing biotin supply.

## Methods

### Strains, growth medium and culture conditions

Strains used in this study are listed in Table 5. The original strain *C. glutamicum* JL-6 (*C. glutamicum* AEC^r^ SD^r^ FP^s^ Met^l^), which is resistant to *S*-2-aminoethyl-l-cysteine (AEC^r^) and sulfadiazine (SD^r^), and is sensitive to *β*-fluoro-pyruvate (FP^s^) as well as an leaky mutant for l-methionine (Met^l^), was derived from the wild-type strain *C. glutamicum* ATCC 13032 after multiple rounds of random mutagenesis. This strain has been deposited in the China Information Center of Industrial Microbial, and the number is CICIM B1031.

**Table 5:** Strains used in this study

Strains	Relevant characteristic(s)	Reference
*E. coli* JM109	*recA*1 *endA*1 *gyrA*96 *thi*-1 *hsdR*17 *e*14-(*mcrA*-) *supE*44 *relA*1 ∆(*lac*-*proAB*)/F’ [*traD*36 *proB *+ *lacl*q lacZ∆M15]	Stratagene
*C. glutamicum* strains		
ATCC13032	Wild-type strain, biotin auxotrophic	ATCC
Lys5	l-lysine producer with the mutation of *pyc* gene	[5]
JL-6 (or CICIM B1031)	*C. glutamicum* AEC^r^ SD^r^ FP^s^ Met^l^, derived from strain ATCC13032, deposited in China Information Center of Industrial Microbial (CICIM)	CICIM
JL-61	Deletion of *pck* gene in strain JL-6 chromosome	This work
JL-62	Deletion of *odx* gene in strain JL-6 chromosome	This work
JL-63	Deletion of *malE* gene in strain JL-6 chromosome	This work
JL-64	Replacement of the natural promoter of *malE* gene with the *tuf* promoter in strain JL-6 chromosome	This work
JL-65	Deletion of *pck* and *odx* genes in strain JL-6 chromosome	This work
JL-66	Replacement of *pck* gene by *ppc* cassette in strain JL-6 chromosome	This work
JL-67	Replacement of *odx* gene by *pyc* cassette in strain JL-6 chromosome	This work
JL-68	Replacement of *pck* and *odx* gene by *ppc* and *pyc* cassette respectively in strain JL-6 chromosome	This work
JL-68∆*gltA*	Deletion of *gltA* gene in strain JL-68 chromosome	This work
JL-68∆*ramA*	Deletion of *ramA* gene in strain JL-68 chromosome	This work
JL-68∆P1*gltA* (or JL-69)	Deletion of 160 bp fragment contained P1 promoter of *gltA* gene in strain JL-68 chromosome	This work
JL-68∆P12*gltA*	Deletion of 540 bp fragment contained P1 and P2 promoter of *gltA* gene in strain JL-68 chromosome	This work
JL-68P_dapA-L1_ *gltA*	Replacement of the natural promoter of *gltA* gene with the *dapA*-*L1* promoter in strain JL-68 chromosome	This work
JL-68∆*prpC1*	Deletion of *prpC1* gene in strain JL-68 chromosome	This work
JL-68 ∆*prpC2*	Deletion of *prpC2* gene in strain JL-68 chromosome	This work
JL-68∆*prpC1*∆*prpC2*	Deletion of *prpC1* and *prpC2* genes in strain JL-68 chromosome	This work
JL-68∆*gltA*∆*prpC1*	Deletion of *prpC1* gene in strain JL-68∆*gltA* chromosome	This work
JL-68∆*gltA*∆*prpC2*	Deletion of *prpC2* gene in strain JL-68∆*gltA* chromosome	This work
JL-68∆*gltA*∆*prpC1*∆*prpC2*	Deletion of *prpC1* and *prpC2* genes in strain JL-68∆*gltA* chromosome	This work
JL-68∆*gltA*∆*prpC1prpR*^G977A^	G977A mutation in *prpR* of JL-67∆*gltA*∆*prpC1* chromosome	This work
JL-69P_dapA-L1_ *gdh*	Replacement of the natural promoter of *gdh* gene with the *dapA*-*L1* promoter in strain JL-69 chromosome	This work
JL-69P_tac_ *gdh*	Replacement of the natural promoter of *gdh* gene with the *tac* promoter in strain JL-69 chromosome	This work
JL-69P_tac-M_ *gdh*	Replacement of the natural promoter of *gdh* gene with the *tac*-*M* promoter in strain JL-69 chromosome	This work
JL-69P_tuf_ *gdh*	Replacement of the natural promoter of *gdh* gene with the *tuf* promoter in strain JL-69 chromosome	This work
JL-69P_sod_ *gdh*	Replacement of the natural promoter of *gdh* gene with the *sod* promoter in strain JL-69 chromosome	This work

The growth medium and culture conditions were illustrated in Additional file 1.

### Construction of *C. glutamicum* recombinant strains

The gene deletions and gene replacements executed in *C. glutamicum* chromosome were performed according to the published method [1]. The recombinant plasmids were transferred into *C. glutamicum* competent cell by electroporation, and the recombinant strains were screened on the LBHIS agar plates containing 25 µg mL^−1^ of Km [42]. The second round of positive selection was carried out by using sucrose as selected marker. The DNA manipulations and build process of the recombinant strain are stated in Additional file 1. The gene deletions were verified by PCR analysis using relevant primers according to the description of Additional file 1: Table S2. The gene replacements were validated via sequencing by Sangon Biotech (Shanghai) Co., Ltd. (Shanghai, China).

### RNA isolation and quantitative real-time PCR (qRT-PCR)

Total cellular RNA was extracted from cells at the exponential phase with the total RNA extraction kit (BioFlux, Beijing, China) as described by the manufacturer. To eliminate residual DNA, RNA preparations were treated with DNase I. The cDNA was synthesized using RevertAid™ First Strand cDNA synthesis kit (Fermentas, Shanghai, China). The qRT-PCR was performed using the QIAGEN OneStep RT-PCR Kit (TIANGEN, Beijing, China) on an iCycler iQ5 real-time PCR system (Bio-Rad, Richmond, USA). RNA (1 μg) and 0.6 μmol L^−1^ of each primer (final concentration) were added to the RT-PCR mixture (50 μL), and the primers used for qRT-PCR are listed in Additional file 1: Table S2. The PCR procedure was prepared following the instructions of the kit (TIANGEN, Beijing, China). The target gene transcriptional levels were normalized to the 16S rRNA from the same RNA samples. Each sample was analyzed in triplicate.

### Analytical methods

A sample was taken from the shake flasks or fermenter every 2 or 4 h. A half of sample was used to measure the biomass using a spectrophotometer at 600 nm after an appropriate dilution. According to the previous description [1], the correlation factor between dry cell weight (DCW) and OD_600_ was determined as 0.318 (1 OD_600_ = 0.318 g DCW). The other half of sample was diluted 100-fold, and then used to determine the glucose and l-lysine concentration using an SBA-40E immobilized enzyme biosensor (Shandong, China). In addition, the samples were also used to determine the concentration of by-products (including amino acids and organic acids) by high performance liquid chromatography (HPLC) according to the description of Xu et al. [5]. The enzyme activity assay is stated in Additional file 1.

## Additional file

**Additional file 1.** Additional materials.

## Abbreviations

TCA: tricarboxylic acid
PEP: phosphoenolpyruvate
OAA: oxaloacetate
α-KG: α-ketoglutarate
PFAAs: pyruvate-family amino acids
DCW: dry cell weight
α: glucose conversion efficiency
μ_max_: maximal specific growth rate
PEPCx: PEP carboxylase
PEPCk: PEP carboxykinase
PCx: pyruvate carboxylase
ODx: OAA decarboxylase
MQO: malate:quinone oxidoreductase
GDH: glutamate dehydrogenase
CS: citrate synthase
MCS: 2-methylcitrate synthases
