# Supplementary material for: Rational modification of tricarboxylic acid cycle for improving l-lysine production in Corynebacterium glutamicum
Source: Microb Cell Fact. 2018 Jul 7;17:105. doi: 10.1186/s12934-018-0958-z (PMC6035423; doi:10.1186/s12934-018-0958-z)
Supplement: Supplementary file 1 — Additional file 1. Additional materials. [file 12934_2018_958_MOESM1_ESM.docx]

**Rational modification of tricarboxylic acid cycle for improving L-lysine production in *Corynebacterium glutamicum***

**Jian-Zhong Xu ^*^**

The Key Laboratory of Industrial Biotechnology, Ministry of Education, School of Biotechnology, Jiangnan University, 1800^#^ Lihu Road, WuXi 214122, People’s Republic of China; E-mail: xujianzhong@jiangnan.edu.cn

**Ze-Hua Wu**

Research and Development Department, Shandong Shouguang Juneng Golden Corn Co., Ltd., 1199^#^ Xinxing Street, Shouguang 262700, People’s Republic of China; E-mail: aixuexi_2016@163.com

**Shi-Jun Gao ^*^**

Research and Development Department, Shandong Shouguang Juneng Golden Corn Co., Ltd., 1199^#^ Xinxing Street, Shouguang 262700, People’s Republic of China; E-mail: jnjym168@126.com

**Weiguo Zhang**

The Key Laboratory of Industrial Biotechnology, Ministry of Education, School of Biotechnology, Jiangnan University, 1800^#^ Lihu Road, WuXi 214122, People’s Republic of China; E-mail: zwgjnedu@sina.cn

^*^ Corresponding authors:

Jian-Zhong Xu; E-mail: [xujianzhong@jiangnan.edu.cn](mailto:xujianzhong@jiangnan.edu.cn); Tel: +86-510-85329312; Fax: +86-510-85329312

Shi-Jun Gao; E-mail: [jnjym168@126.com](mailto:jnjym168@126.com); Tel: +86-536-5185080; Fax: +86-536-5185678

**Growth medium and culture conditions**

Luria-Bertani (LB) and LBG (LB supplemented with 5 g L^-1^ glucose) were used as standard media for *Escherichia coli* and *C. glutamicum*, respectively [[1](#_ENREF_1)]. Epo medium used for preparing electroporation-competent *C. glutamicum* cells and LBHIS (LB, Brain Heart Infusion, and sorbitol) medium used for obtaining recombinant strains were prepared according to the description of van der Rest *et al.* [[2](#_ENREF_2)]. *C. glutamicum* and *E. coli* were grown at 30 °C and 37 °C, respectively. When appropriate, *E. coli* was incubated with 50 µg mL^-1^ of kanamycin (Km), and 25 µg mL^-1^ of Km was used to obtain recombinant strains of *C. glutamicum*. Isopropyl-β-D-thio-galactoside (IPTG) was added at a final concentration of 1 mmol L^-1^ for gene expression [[3](#_ENREF_3)].

Batch cultivation in shake flasks was carried out as described previously by Xu *et al.* [[4](#_ENREF_4)]. The improved CgXIIM-medium (CgXII medium supplied with 0.25 g L^-1^ l-methionine) without a carbon source was used as minimal medium for L-lysine production. The main culture was performed in triplicate using 500-mL Erlenmeyer flasks with 50 mL of CgXIIM-medium containing 40 g L^-1^ of glucose.

The fed-batch fermentations were carried out in a 5-L jar fermenter (BLBio-5GJ-2-H, Bailun Bi-Technology Co. Ltd., Shanghai, China) containing 1 L of medium with an inoculum size of 10% (v/v) from a seed culture grown to ∆OD_562_=0.45-0.50 (at a dilution of 25-fold). Culture medium used for seed culture consisted of (per liter): 25 g glucose, 30 g corn steep liquor, 5 g (NH_4_)_2_SO_4_, 1 g KH_2_SO_4_, 0.5 g MgSO_4_∙7H_2_O, 0.25 g L-methionine and 10 g CaCO_3_. The fermentation medium contained (per liter): 80 g glucose, 40 g beet molasses, 30 g corn steep liquor, 50 g (NH_4_)_2_SO_4_, 1.5 g KH_2_SO_4_, 1.0 g MgSO_4_∙7H_2_O, 0.02 g FeSO_4_, 0.02 g MnSO_4_, 0.5 g l-methionine, 0.05 g glycine betaine, 400 μg thiamine-HCl, 800 μg biotin and 2 mL antifoam. The aeration rate, pH, dissolved oxygen levels, and temperature were set according to the reports by Becker *et al.* [[5](#_ENREF_5)]. Feed solution, containing 800 g L^-1^ sterile glucose and 400 g L^-1^ (NH_4_)_2_SO_4_, was used to maintain the glucose concentration at 20~30 g L^-1^ by adjusting the feeding rate according to the glucose concentration checked every 4 h. Both these media were adjusted to pH 7.0 with ammonium hydroxide. When appropriate, 0.6 g L^-1^ of sterile biotin solution was used to regulate the biotin concentration.

**DNA manipulations and transformations**

The plasmids and oligonucleotides used in this study are listed in Table S1 and Table S2, respectively. Chromosomal DNA was extracted from *C. glutamicum* using an Ezup Bacterial Genomic DNA Extraction Kit according the protocol supplied by the manufacturer (Sangon, Shanghai, China). The target gene segments were amplified using Phusion high-fidelity DNA polymerase (Finnzyme, Espoo, Finland) from *C. glutamicum* chromosomal DNA using corresponding primers (Table S2). The plasmid construction and transformation were performed according to the descriptions of previous reports [[3](#_ENREF_3)]. The build processes of plasmids and strains are illustrated as follows. Plasmids were extracted from *E. coli* using the SanPrep Mini Plasmid Kit (Sangon, Shanghai, China) and confirmed by restriction endonuclease reaction.

**Enzyme activity assay**

The crude enzyme was prepared according to the method reported by Xu *et al.* [[6](#_ENREF_6)]. After centrifugation at 4°C for 30 min at 10000 *g*, the cell-free supernatants were immediately used to determine the enzyme activities. Protein concentrations were determined using the Bradford Protein Quantification Kit (Sangon, Shanghai, China) with bovine serum albumin as standard. The analyses of enzyme activities and protein concentrations were done in triplicate. Specific activity was given as the number of U mg^-1^of protein. The CS, MCS1 and MCS2 enzymes analysis was based on the protocol of Claes *et al.* [[7](#_ENREF_7)]. Assay for aspartate aminotransferase (AAT)[[8](#_ENREF_8)] and GDH [[9](#_ENREF_9)] were performed as previously described. The phosphoenolpyruvate carboxylase (PEPCx), phosphoenolpyruvate carboxykinase (PEPCk), pyruvate carboxylase (PCx) and malate dehydrogenase (MDH) enzymes analysis was based on the protocol of Xu *et al.* [4]. The pyruvate kinase (PK), oxaloacetate decarboxylase (ODx) and malic enzyme (MalE) enzymes analysis was based on the protocol of Jetten *et al.* [[10](#_ENREF_10)]. Assay for malate:quinone oxidoreductase (MQO) were performed as previously described by Mitsuhashi *et al.* [[11](#_ENREF_11)].

**The procedures of integration vectors constructions**

The integration vectors pK18*-*MBPMT/*∆pck* and pK18*-*MBPMT/*∆pck::ppc* construction

The *pck* left (*pck*-L) and right (*pck*-R) arms from *C. glutamicum* JL-6 were amplified by PCR with the corresponding primer pairs, respectively (Table S2). The resulting fragments were purified by the SanPrep DNA Gel Extraction Kit. The fragments of *pck*-L/*pck*-R were purified and digested by suitable restriction enzyme (Table S2), respectively, and then were orderly ligated into pK18*-*MBPMT which was similarly digested. The resulting plasmid was designated as pK18*-*MBPMT/*∆pck.*

For construction of pK18*-*MBPMT/*∆pck::ppc*, DNA fragment of *ppc* gene was amplified with high-fidelity *pyrobest* DNA polymerase (TAKARA, Dalian, China) from the chromosomal DNA of *C. glutamicum* ATCC13032 by *ppc*-F/*ppc*-R (Upstream primer contains an SD sequence). The fragment was purified and digested by suitable restriction enzyme, and then was ligated into pK18*-*MBPMT/*∆pck* which was similarly digested (Table S2), then the resulting plasmid was designated as pK18*-*MBPMT/*∆pck::ppc.*

The integration vectors pK18*-*MBPMT/*∆odx* and pK18*-*MBPMT/*∆odx::pyc* construction

The *odx* left (*odx*-L) and right (*odx*-R) arms from *C. glutamicum* JL-6 were amplified by PCR with the corresponding primer pairs, respectively (Table S2). The resulting fragments were purified by the SanPrep DNA Gel Extraction Kit. The fragments of *odx*-L/*odx*-R were purified and digested by suitable restriction enzyme (Table S2), respectively, and then were orderly ligated into pK18*-*MBPMT which was similarly digested. The resulting plasmid was designated as pK18*-*MBPMT/*∆odx.*

For construction of pK18*-*MBPMT/*∆odx::pyc*, DNA fragment of *pyc* gene was amplified with high-fidelity *pyrobest* DNA polymerase (TAKARA, Dalian, China) from the chromosomal DNA of *C. glutamicum* Lys5 by *pyc*-F/*pyc*-R (Upstream primer contains an SD sequence). The fragment was purified and digested by suitable restriction enzyme, and then was ligated into pK18*-*MBPMT/*∆odx* which was similarly digested (Table S2), then the resulting plasmid was designated as pK18*-*MBPMT/*∆odx::pyc.*

The integration vector pK18*mobsacB-∆malE* construction

The *malE* left (*malE*-L) and right (*malE*-R) arms from *C. glutamicum* JL-6 were amplified by PCR with the corresponding primer pairs, respectively. The resulting fragments were purified by the SanPrep DNA Gel Extraction Kit. The fragments of *malE*-L/*malE*-R were purified and digested by suitable restriction enzyme (Table S2), respectively, and then were orderly ligated into pK18*mobsacB*, resulting in plasmid pK18*mobsacB*-*∆malE*.

The integration vector pK18*mobsacB-*P_tuf_ *malE* construction

The building process was referred to Figure S6. In PCR1, the upstream sequence of the *malE* gene (i.e., *malE*_Up_) was amplified using primers *malE*_Up_-F and *malE*_Up_-P_tuf_ -R, whereby an overlapping sequence with the *tuf* promoter was artificially added at the 3’-end. In PCR2, the *tuf*-promoter was amplified using primers P*tuf_malE_*-F and P*tuf_malE_*-R, whereby an overlapping sequence with the *malE* gene was artificially added at the 3’-end. In PCR3, the *malE* operon was amplified using primers *malE-*F and *malE-*R. In the next step, two DNA fragments from PCR2 and PCR3 are fused in PCR4 with the *tuf*-promoter and *malE* operon specific primer sequences used in PCR2 and PCR3, respectively and cleaned DNA from PCR2 and PCR3. Whereafter, the DNA-fragments from PCR1 and PCR4 are fused by PCR5 using cleaned DNA from PCR1 and PCR4 as template DNA and primers *malE*_Up_-F and *malE-*R. Recognition sites for *Eco*RI and *Hin*dIII were used for vector-insert-ligation. The fragment was purified and digested by *Eco*RI and *Hin*dIII, and then was ligated into pK18*mobsacB* which was similarly digested, then the resulting plasmid was designated as pK18*mobsacB-*P_tuf_ *malE.*

The integration vector pK18*mobsacB-∆gltA* construction

The *gltA* left (*gltA*-L) and right (*gltA*-R) arms from *C. glutamicum* JL-6 were amplified by PCR with the corresponding primer pairs, respectively. The resulting fragments were purified by the SanPrep DNA Gel Extraction Kit. The fragments of *gltA*-L/*gltA*-R were purified and digested by suitable restriction enzyme (Table S2), respectively, and then were orderly ligated into pK18*mobsacB*, resulting in plasmid pK18*mobsacB*-*∆gltA.*

The integration vector pK18*mobsacB-∆ramA* construction

The *ramA* left (*ramA*-L) and right (*ramA*-R) arms from *C. glutamicum* JL-6 were amplified by PCR with the corresponding primer pairs, respectively. The resulting fragments were purified by the SanPrep DNA Gel Extraction Kit. The fragments of *ramA*-L/*ramA*-R were purified and digested by suitable restriction enzyme (Table S2), respectively, and then were orderly ligated into pK18*mobsacB*, resulting in plasmid pK18*mobsacB*-*∆ramA.*

The integration vector pK18*mobsacB-∆*P1*gltA* construction

The *gltA*_P1_ left (*gltA*_P1_-L) and right (*gltA*_P1_-R) arms from *C. glutamicum* JL-6 were amplified by PCR with the corresponding primer pairs, respectively. The resulting fragments were purified by the SanPrep DNA Gel Extraction Kit. The fragments of *gltA*_P1_-L/*gltA*_P1_-R were purified and digested by suitable restriction enzyme (Table S2), respectively, and then were orderly ligated into pK18*mobsacB*, resulting in plasmid pK18*mobsacB*-*∆*P1*gltA.*

The integration vector pK18*mobsacB-∆*P12*gltA* construction

The *gltA*_P12_ left (*gltA*_P12_-L) and right (*gltA*_P12_-R) arms from *C. glutamicum* JL-6 were amplified by PCR with the corresponding primer pairs, respectively. The resulting fragments were purified by the SanPrep DNA Gel Extraction Kit. The fragments of *gltA*_P12_-L/*gltA*_P12_-R were purified and digested by suitable restriction enzyme (Table S2), respectively, and then were orderly ligated into pK18*mobsacB*, resulting in plasmid pK18*mobsacB*-*∆*P12*gltA.*

The integration vector pK18*mobsacB*-*prpR*^G977A^ construction

A part of the *prpR* gene from *C. glutamicum* JL-6, including the mutation region was amplified amplified by PCR with primer pair *prpR*-F/*prpR*-R. The resulting fragments were purified by the SanPrep DNA Gel Extraction Kit, and ligated into pMD^TM^ 18T vector via T-A cloning resulting in pMD^TM^ 18T-*prpR*. The G→A mutation of fragment *prpR* at loci 977 was carried out by overlap extension PCR with plasmid pMD^TM^ 18T-*prpR* as template and *MR*-F/*MR*-R as primer. Mutation positive strains were screened and confirmed by gene sequencing. The mutation positive plasmid was extracted from positive strain, and digested with *Xba*I/*Sal*I, and ligated into plasmid pK18*mobsacB*, resulting in plasmid pK18*mobsacB-prpR*^G977A^.

The integration vector pK18*mobsacB-∆prpC1* construction

The *prpC1* left (*prpC1*-L) and right (*prpC1*-R) arms from *C. glutamicum* JL-6 were amplified by PCR with the corresponding primer pairs, respectively. The resulting fragments were purified by the SanPrep DNA Gel Extraction Kit. The fragments of *prpC1*-L/*prpC1*-R were purified and digested by suitable restriction enzyme (Table S2), respectively, and then were orderly ligated into pK18*mobsacB*, resulting in plasmid pK18*mobsacB*-*∆prpC1.*

The integration vector pK18*mobsacB-∆prpC2* construction

The *prpC2* left (*prpC2*-L) and right (*prpC2*-R) arms from *C. glutamicum* JL-6 were amplified by PCR with the corresponding primer pairs, respectively. The resulting fragments were purified by the SanPrep DNA Gel Extraction Kit. The fragments of *prpC2*-L/*prpC2*-R were purified and digested by suitable restriction enzyme (Table S2), respectively, and then were orderly ligated into pK18*mobsacB*, resulting in plasmid pK18*mobsacB*-*∆prpC2.*

The integration vector pK18*mobsacB-*P_dapA-L1_ *gltA* construction

The building process was referred to Figure S6. In PCR1, the upstream sequence of the *gltA* gene (i.e., *gltA*_Up_) was amplified using primers *gltA*_Up_-F and *gltA*_Up_-P_dapA_-R, whereby an overlapping sequence with the *dapA-L1* promoter was artificially added at the 3’-end. In PCR2, the *dapA-L1* promoter was amplified using primers P*dapA_gltA_*-F and P*dapA_gltA_*-R, whereby an overlapping sequence with the *gltA* gene was artificially added at the 3’-end. In PCR3, the *gltA* operon was amplified using primers *gltA-*F and *gltA-*R. In the next step, two DNA fragments from PCR2 and PCR3 are fused in PCR4 with the *dapA-L1* promoter and *gltA* operon specific primer sequences used in PCR2 and PCR3, respectively and cleaned DNA from PCR2 and PCR3. Whereafter, the DNA-fragments from PCR1 and PCR4 are fused by PCR5 using cleaned DNA from PCR1 and PCR4 as template DNA and primers *gltA*_Up_-F and *gltA-*R. Recognition sites for *Eco*RI and *Hin*dIII were used for vector-insert-ligation. The fragment was purified and digested by *Eco*RI and *Hin*dIII, and then was ligated into pK18*mobsacB* which was similarly digested, then the resulting plasmid was designated as pK18*mobsacB-*P_dapA-L1_ *gltA.*

The integration vector pK18*mobsacB-*P_dapA-L1_ *gdh* construction

The building process was referred to Figure S6. In PCR1, the upstream sequence of the *gdh* gene (i.e., *gdh*_Up_) was amplified using primers *gdh*_Up_-F and *gdh*_Up_-P_dapA_-R, whereby an overlapping sequence with the *dapA-L1* promoter was artificially added at the 3’-end. In PCR2, the *dapA-L1* promoter was amplified using primers P*dapA_gdh_*-F and P*dapA_gdh_*-R, whereby an overlapping sequence with the *gdh* gene was artificially added at the 3’-end. In PCR3, the *gdh* operon was amplified using primers *gdh-*F and *gdh-*R. In the next step, two DNA fragments from PCR2 and PCR3 are fused in PCR4 with the *dapA-L1* promoter and *gdh* operon specific primer sequences used in PCR2 and PCR3, respectively and cleaned DNA from PCR2 and PCR3. Whereafter, the DNA-fragments from PCR1 and PCR4 are fused by PCR5 using cleaned DNA from PCR1 and PCR4 as template DNA and primers *gdh*_Up_-F and *gdh-*R. Recognition sites for *Eco*RI and *Hin*dIII were used for vector-insert-ligation. The fragment was purified and digested by *Eco*RI and *Hin*dIII, and then was ligated into pK18*mobsacB* which was similarly digested, then the resulting plasmid was designated as pK18*mobsacB-*P_dapA-L1_ *gdh.*

The integration vector pK18*mobsacB-*P_tac_ *gdh* and pK18*mobsacB-*P_tac-M_ *gdh* construction

The building process was referred to Figure S6. In PCR1, the upstream sequence of the *gdh* gene (i.e., *gdh*_Up_) was amplified using primers *gdh*_Up_-F and *gdh*_Up_-P_tac/M_-R, whereby an overlapping sequence with the *tac/tac-M* promoter was artificially added at the 3’-end. In PCR2, the *tac/tac-M* promoter was amplified using primers P*tac/M_gdh_*-F and P*tac/M_gdh_*-R, whereby an overlapping sequence with the *gdh* gene was artificially added at the 3’-end. In PCR3, the *gdh* operon was amplified using primers *gdh-*F and *gdh-*R. In the next step, two DNA fragments from PCR2 and PCR3 are fused in PCR4 with the *tac/tac-M* promoter and *gdh* operon specific primer sequences used in PCR2 and PCR3, respectively and cleaned DNA from PCR2 and PCR3. Whereafter, the DNA-fragments from PCR1 and PCR4 are fused by PCR5 using cleaned DNA from PCR1 and PCR4 as template DNA and primers *gdh*_Up_-F and *gdh-*R. Recognition sites for *Eco*RI and *Hin*dIII were used for vector-insert-ligation. The fragment was purified and digested by *Eco*RI and *Hin*dIII, and then was ligated into pK18*mobsacB* which was similarly digested, then the resulting plasmid was designated as pK18*mobsacB-*P_tac_ *gdh* or pK18*mobsacB-*P_tac-M_ *gdh.*

The integration vector pK18*mobsacB-*P_tuf_ *gdh* construction

The building process was referred to Figure S6. In PCR1, the upstream sequence of the *gdh* gene (i.e., *gdh*_Up_) was amplified using primers *gdh*_Up_-F and *gdh*_Up_-P_tuf_-R, whereby an overlapping sequence with the *tuf* promoter was artificially added at the 3’-end. In PCR2, the *tuf* promoter was amplified using primers P*tuf_gdh_*-F and P*tuf_gdh_*-R, whereby an overlapping sequence with the *gdh* gene was artificially added at the 3’-end. In PCR3, the *gdh* operon was amplified using primers *gdh-*F and *gdh-*R. In the next step, two DNA fragments from PCR2 and PCR3 are fused in PCR4 with the *tuf* promoter and *gdh* operon specific primer sequences used in PCR2 and PCR3, respectively and cleaned DNA from PCR2 and PCR3. Whereafter, the DNA-fragments from PCR1 and PCR4 are fused by PCR5 using cleaned DNA from PCR1 and PCR4 as template DNA and primers *gdh*_Up_-F and *gdh-*R. Recognition sites for *Eco*RI and *Hin*dIII were used for vector-insert-ligation. The fragment was purified and digested by *Eco*RI and *Hin*dIII, and then was ligated into pK18*mobsacB* which was similarly digested, then the resulting plasmid was designated as pK18*mobsacB-*P_tuf_ *gdh.*

The integration vector pK18*mobsacB-*P_sod_ *gdh* construction

The building process was referred to Figure S6. In PCR1, the upstream sequence of the *gdh* gene (i.e., *gdh*_Up_) was amplified using primers *gdh*_Up_-F and *gdh*_Up_-P_sod_-R, whereby an overlapping sequence with the *sod* promoter was artificially added at the 3’-end. In PCR2, the *sod* promoter was amplified using primers P*sod_gdh_*-F and P*sod_gdh_*-R, whereby an overlapping sequence with the *gdh* gene was artificially added at the 3’-end. In PCR3, the *gdh* operon was amplified using primers *gdh-*F and *gdh-*R. In the next step, two DNA fragments from PCR2 and PCR3 are fused in PCR4 with the *sod* promoter and *gdh* operon specific primer sequences used in PCR2 and PCR3, respectively and cleaned DNA from PCR2 and PCR3. Whereafter, the DNA-fragments from PCR1 and PCR4 are fused by PCR5 using cleaned DNA from PCR1 and PCR4 as template DNA and primers *gdh*_Up_-F and *gdh-*R. Recognition sites for *Eco*RI and *Hin*dIII were used for vector-insert-ligation. The fragment was purified and digested by *Eco*RI and *Hin*dIII, and then was ligated into pK18*mobsacB* which was similarly digested, then the resulting plasmid was designated as pK18*mobsacB-*P_sod_ *gdh.*

**The procedures of recombinant strain constructions**

Construction of *C. glutamicum* JL-61, *C. glutamicum* JL-62, *C. glutamicum* JL-63, *C. glutamicum* JL-64, *C. glutamicum* JL-66, and *C. glutamicum* JL-67

The plasmid pK18*-*MBPMT/*∆pck*, pK18*-*MBPMT/*∆odx*, pK18*mobsacB*-*∆malE*, pK18*mobsacB-*P_tuf_ *malE*, pK18*-*MBPMT/*∆pck::ppc* and pK18*-*MBPMT/*∆odx::pyc* was transformed into *C. glutamicum* JL-6, respectively, then the resulting recombinant strain was designated as *C. glutamicum* JL-61, *C. glutamicum* JL-62, *C. glutamicum* JL-63, *C. glutamicum* JL-64, *C. glutamicum* JL-66, and *C. glutamicum* JL-67, respectively.

Construction of *C. glutamicum* JL-65

The plasmid pK18*-*MBPMT/*∆odx* was transformed into *C. glutamicum* JL-61 resulting in *C. glutamicum* JL-65.

Construction of *C. glutamicum* JL-68

The plasmid pK18*-*MBPMT/*∆odx::pyc* was transformed into *C. glutamicum* JL-66 resulting in *C. glutamicum* JL-68.

Construction of *C. glutamicum* JL-68∆*gltA*, *C. glutamicum* JL-68∆*ramA*, *C. glutamicum* JL-68∆P1*gltA* (or JL-69), *C. glutamicum* 68∆P12*gltA*, and *C. glutamicum* JL-68P_dapA-L1_ *gltA*

The plasmid pK18*mobsacB*-*∆gltA*, pK18*mobsacB*-*∆ramA*, pK18*mobsacB*-*∆*P1*gltA*, pK18*mobsacB*-*∆*P12*gltA* and pK18*mobsacB-*P_dapA-L1_ *gltA* was transformed into *C. glutamicum* JL-68, respectively, then the resulting recombinant strain was designated as *C. glutamicum* JL-68∆*gltA*, *C. glutamicum* JL-68∆*ramA*, *C. glutamicum* JL-68∆P1*gltA* (or JL-69), *C. glutamicum* 68∆P12*gltA*, and *C. glutamicum* JL-68P_dapA-L1_ *gltA*, respectively*.*

Construction of *C. glutamicum* JL-68∆*gltA*∆*prpC1* and *C. glutamicum* JL-68∆*gltA*∆*prpC2*

The plasmid pK18*mobsacB*-*∆prpC1* and pK18*mobsacB*-*∆prpC2* was transformed into *C. glutamicum* JL-68∆*gltA*, respectively, and then the resulting recombinant strain was designated as *C. glutamicum* JL-68∆*gltA*∆*prpC1* and *C. glutamicum* JL-68∆*gltA*∆*prpC2*, respectively.

Construction of *C. glutamicum* JL-68∆*gltA*∆*prpC1*∆*prpC2* and *C. glutamicum* JL-68∆*gltA*∆*prpC1prpR*^G977A^

The plasmid pK18*mobsacB*-*∆prpC2* and pK18*mobsacB*-*prpR*^G977A^ was transformed into *C. glutamicum* JL-68∆*gltA*∆*prpC1*, respectively, and then the resulting recombinant strain was designated as *C. glutamicum* JL-68∆*gltA*∆*prpC1*∆*prpC2* and *C. glutamicum* JL-68∆*gltA*∆*prpC1prpR*^G977A^, respectively.

Construction of *C. glutamicum* JL-69P_dapA-L1_ *gdh*, *C. glutamicum* JL-69P_tac_ *gdh*, *C. glutamicum* JL-69P_tac-M_ *gdh*, *C. glutamicum* JL-69P_tuf_ *gdh*, and *C. glutamicum* JL-69P_sod_ *gdh*

The plasmid pK18*mobsacB*-P_dapA-L1_ *gdh*, pK18*mobsacB*-P_tac_ *gdh*, pK18*mobsacB*-P_tac-M_ *gdh*, pK18*mobsacB*-P_tuf_ *gdh* and pK18*mobsacB-*P_sod_ *gdh* was transformed into *C. glutamicum* JL-68∆P1*gltA* (or JL-69), respectively, then the resulting recombinant strain was designated as *C. glutamicum* JL-69P_dapA-L1_ *gdh*, *C. glutamicum* JL-69P_tac_ *gdh*, *C. glutamicum* JL-69P_tac-M_ *gdh*, *C. glutamicum* JL-69P_tuf_ *gdh*, and *C. glutamicum* JL-69P_sod_ *gdh*, respectively*.*

**The effect of malic enzyme on L-lysine productivity**

Malic enzyme (MalE, encoded by *malE* gene), a key enzyme in PEP-pyruvate-OAA node, catalyzes reversible interconversion of malate and pyruvate [[12](#_ENREF_12)]. MalE can be used to maintain the NADPH balance, especially during growth on gluconeogenic substrates [[13](#_ENREF_13), [14](#_ENREF_14)] because MalE from *C. glutamicum* is a strictly NADP^+^-dependent version [[15](#_ENREF_15)]. However, the effect of MalE on L-lysine production has always been controversial. Some researches indicated that overexpression of MalE is beneficial to increase L-lysine production [[13](#_ENREF_13), [16](#_ENREF_16), [17](#_ENREF_17)], whereas the others asserted that it has no effect on L-lysine production by *C. glutamicum* [[18](#_ENREF_18), [19](#_ENREF_19)]. To test the effect of MalE on lysine production in *C. glutamicum* JL-6, we constructed *malE*-deficient strain *C. glutamicum* JL-63 and *malE*-overexpressing strains *C. glutamicum* JL-64, and determined the specific activities of MalE as well as the accumulation of L-lysine and by-products in these strains. *C. glutamicum* JL-64 showed specific activities of MalE of 5.07±0.44 U (mg of protein)^-1^, whereas *C. glutamicum* JL-63 contains no detectable MalE (Table S3). However, a comparative analysis of *C. glutamicum* JL-6, JL-63 and JL-64 revealed that genetic modification of MalE did not lead to significant changes of L-lysine production (Table S4). These results are in accordance with the reports given by Netzer *et al.* [[18](#_ENREF_18)] and Georgi *et al.* [[19](#_ENREF_19)]. The concentration of L-lysine was similar in *C. glutamicum* JL-6, *C. glutamicum* JL-63, and *C. glutamicum* JL-64, i.e., 14.47±0.52, 14.51±0.97 and 14.21±0.52 g L^-1^, respectively. In addition, the *q*_Lys, max_ of these strains were about 0.25±0.01, 0.24±0.02 and 0.22±0.03 g g^-1^ h^-1^, respectively (Table S4). Moreover, the cell growth and accumulation of by-products were also not significantly changed during modification of *malE* gene. Therefore, MalE will probably do little to increase L-lysine production. Although *malE* deletion led to slightly increase the L-lysine production (from 14.47±0.52 g L^-1^ to 14.51±0.97 g L^-1^), we did not intend to delete MalE-coding gene *malE* to improve L-lysine production because MalE participates in maintaining the flexibility and genetic robustness of *C. glutamicum* (Netzer *et al.* 2004).

**Table S1** The plasmids used in this study

| Plasmids | Relevant characteristic(s) | Reference |
| --- | --- | --- |
| pMD^TM^ 18-T Vector | T-A Cloning Kit | Stratagene |
| pDXW-8 | Amp^r^ and Km^r^ *E. coli-C. glutamicum* shuttle vector with *tac* promoter | [[20](#_ENREF_20)] |
| pDXW-10 | Amp^r^ and Km^r^ *E. coli-C. glutamicum* shuttle vector with *tac-M* promoter | [[21](#_ENREF_21)] |
| pK18*mobsacB* | Integration vector | [[22](#_ENREF_22)] |
| pK18-MBPMT | pK18*mobsacB* carrying *tac* promoter and *rrnBT1T2* terminator cartridge with iMCS, a integration vector | [[23](#_ENREF_23)] |
| pK18-MBPMT/∆*pck* | Integration vector for knockout of the *pck* gene | This work |
| pK18-MBPMT/∆*odx* | Integration vector for knockout of the *odx* gene | This work |
| pK18*mobsacB*-∆*malE* | Integration vector for knockout of the *malE* | This work |
| pK18*mobsacB*-P_tuf_ *malE* | Integration vector for replacement of the nature promoter of *malE* gene by the *tuf* promoter | This work |
| pK18-MBPMT/∆*pck::ppc* | Integration vector for replacement of the *pck* gene by the cassette of *ppc* gene | This work |
| pK18-MBPMT/∆*odx::pyc* | Integration vector for replacement of the *odx* gene by the cassette of *pyc* gene | This work |
| pK18*mobsacB*-*∆gltA* | Integration vector for knockout of the *gltA* gene | This work |
| pK18*mobsacB*-*∆ramA* | Integration vector for knockout of the *ramA* gene | This work |
| pK18*mobsacB*-*∆*P1*gltA* | Integration vector for knockout of the P1 promoter of the *gltA* gene | This work |
| pK18*mobsacB*-*∆*P12*gltA* | Integration vector for knockout of the P1 and P2 promoter of the *gltA* gene | This work |
| pK18*mobsacB*-*∆prpC1* | Integration vector for knockout of the *prpC1* gene | This work |
| pK18*mobsacB*-*∆prpC2* | Integration vector for knockout of the *prpC2* gene | This work |
| pK18*mobsacB*-*prpR*^G977A^ | Integration vector for implementation of the mutation G977A into *prpR* gene | This work |
| pK18*mobsacB*-P_dapA-L1_ *gltA* | Integration vector for replacement of the nature promoter of *gltA* gene by the *dapA-L1* promoter | This work |
| pK18*mobsacB*-P_dapA-L1_ *gdh* | Integration vector for replacement of the nature promoter of *gdh* gene by the *dapA-L1* promoter | This work |
| pK18*mobsacB*-P_tac_ *gdh* | Integration vector for replacement of the nature promoter of *gdh* gene by the *tac* promoter | This work |
| pK18*mobsacB*-P_tac-M_ *gdh* | Integration vector for replacement of the nature promoter of *gdh* gene by the *tac-M* promoter | This work |
| pK18*mobsacB*-P_tuf_ *gdh* | Integration vector for replacement of the nature promoter of *gdh* gene by the *tuf* promoter | This work |
| pK18*mobsacB*-P_sod_ *gdh* | Integration vector for replacement of the nature promoter of *gdh* gene by the *sod* promoter | This work |

**Table S2** The oligonucleotides used in this study

| Oligonucleotide | | | | | 5’→3’ sequence ^a^ | | | | Cleavage sites | | | | | | | | Purposes |
| --- | --- | --- | --- | --- | --- | --- | --- | --- | --- | --- | --- | --- | --- | --- | --- | --- | --- |
| *pck*-L-F | CGGAATTCTCGCGAATACTTCAACACTTG | | | | | | | | *Eco*RI | | | | | | | PCR for the *pck* left arm, *pck*-L | |
| *pck-*L-R | TCCCCCGGGGCCCAGTTGTTGGTTGGGC | | | | | | | | *Sma*I | | | | | | |  |  |
| *pck*-R-F | GCTCTAGATGGCGAAGATGGACGCTTCCTG | | | | | | | | *Xba*I | | | | | | | PCR for the *pck* right arm, *pck*-R | |
| *pck-*R-R | CCCAAGCTTGCAGGTCGTGGGGTTTACTG | | | | | | | | *Hin*dIII | | | | | | |  |  |
| *pck-*F | ATGACTACTGCTGCAATC | | | | | | | | *-* | | | | | | | Verifying the ∆*pck* deletion or *ppc* insertion | |
| *pck*-R | TAAGCGTGAGCTGCTG | | | | | | | | *-* | | | | | | |  |  |
| *ppc-*F | CCGGCGGCCGC*GAAAGGAGATATACC*ATGACTGATTTTTTACGCG | | | | | | | | *Not*I | | | | | | | PCR for the *ppc* operon | |
| *ppc*-R | GGAATTCCATATGTACTTCCGTATCTGGGATCAG | | | | | | | | *Nde*I | | | | | | |  |  |
| *odx*-L-F | TCCCCCGGGCACCGGCATCAAATTGTGTC | | | | | | | | *Sma*I | | | | | | | PCR for the *odx* left arm, *odx*-L | |
| *odx-*L-R | GCGGATCCCACGGGCGGTGAGGTTAG | | | | | | | | *Bam*HI | | | | | | |  |  |
| *odx*-R-F | GCTCTAGACATCGGCAAGCTGGGCAAC | | | | | | | | *Xba*I | | | | | | | PCR for the *odx* right arm, *odx*-R | |
| *odx-*R-R | CCCAAGCTTTTGCCTTGAGCACAATGTC | | | | | | | | *Hin*dIII | | | | | | |  |  |
| *odx-*F | ATGTCGGTCATGCGTTTTG | | | | | | | | *-* | | | | | | | Verifying the ∆*odx* deletion or *pyc* insertion | |
| *odx*-R | TTAGGCGTCCACGACTC | | | | | | | | *-* | | | | | | |  |  |
| *pyc-*F | AAAAGTACT*GAAAGGAGATATACC*ATGTCGACTCACACATCTTC | | | | | | | | *Sca*I | | | | | | | PCR for the *pyc* operon | |
| *pyc*-R | GGAATTCCATATGTTAGGAAACGACGACGATC | | | | | | | | *Nde*I | | | | | | |  |  |
| *malE*-L-F | CGGAATTCGCTGCGTGGAAGTGTTCACAC | | | | | | | | *Eco*RI | | | | | | | PCR for the *malE* left arm, *malE*-L | |
| *malE-*L-R | CGGGATCCTGAGCCTTGCCCTCCATGACG | | | | | | | | *Bam*HI | | | | | | |  |  |
| *malE*-R-F | GCTCTAGACCAGAGATCGATCCTGAGCTG | | | | | | | | *Xba*I | | | | | | | PCR for the *malE* right arm, *malE*-R | |
| *malE-*R-R | CCCAAGCTTCTGAACACTTGTGGCGCGGCG | | | | | | | | *Hin*dIII | | | | | | |  |  |
| *malE-*F | **ATGACCATCGACCTGCAGCGTTCCAC** | | | | | | | | *-* | | | | | | | Verifying the ∆*malE* deletion and *malE* operon | |
| *malE*-R | CCCAAGCTTTTAAGCGTTTTGCGCTTCG | | | | | | | | *Hin*dIII | | | | | | |  |  |
| *malE*_Up_-F | CGGAATTCGCTGCGTGGAAGTGTTCACAC | | | | | | | | *Eco*RI | | | | | | | PCR for the *malE* up segment, *malE*_Up_ | |
| *malE*_Up_-P_tuf_ -R | | | **CACTTACCCTACGCGCCTACTGACACGCT**ATCATTTAGCCTTGTTAATC | | | | | | *-* | | | | | | |  |  |
| P*tuf_malE_*-F | **AGCGTGTCAGTAGGCGCGTAGGGTAAGTG** | | | | | | | | *-* | | | | | | | PCR for the *tuf* promoter, P_tuf_ used for *malE* modification | |
| P*tuf_malE_*-R | **GTGGAACGCTGCAGGTCGATGGTCAT**TGTATGTCCTCCTGGAG | | | | | | | | *-* | | | | | | |  |  |
| *gltA*-L-F | CGGAATTCACTCAGAGGGCAGGGTG | | | | | | | | *Eco*RI | | | | | | | PCR for the *gltA* left arm, *gltA*-L | |
| *gltA*-L-R | CGGGATCCGATGTAGGTGATCTTCG | | | | | | | | *Bam*HI | | | | | | |  |  |
| *gltA*-R-F | CGGGATCCTTCACCGTATTGTTCGC | | | | | | | | *Bam*HI | | | | | | | PCR for the *gltA* right arm, *gltA*-R | |
| *gltA*-R-R | CCCAAGCTTTTGGCGGTGGCGCGCTG | | | | | | | | *Hin*dIII | | | | | | |  |  |
| *gltA-*F | **ATGTTTGAAAGGGATATCGTGGCTACTG** | | | | | | | | *-* | | | | | | | Verifying the ∆*malE* deletion and *gltA* operon | |
| *gltA-*R | CCCAAGCTTTTAGCGCTCCTCGCGAG | | | | | | | | *Hin*dIII | | | | | | |  |  |
| *^*^gltA*_P1_-L-F | CGGAATTCCTGACCCAACAACTATAACCCTGAAGC | | | | | | | | *Eco*RI | | | | | | | PCR for the *gltA*_P1_ left arm, *gltA*_P1_-L | |
| ^*^*gltA*_P1_-L-R | CGGGATCCAGCCAATTCCCCCACAATCACGTTGG | | | | | | | | *Bam*HI | | | | | | |  |  |
| ^*^*gltA*_P1_-R-F | CGGGATCCTCCGAACAAATATGTTTGAAAGGG | | | | | | | | *Bam*HI | | | | | | | PCR for *gltA*_P1_ right arm, *gltA*_P1_-R | |
| *^*^gltA*_P1_-R-R | CCCAAGCTTTGTGCCTCATCGAGTGGGTTCAGC | | | | | | | | *Hin*dIII | | | | | | |  |  |
| *^*^gltA*_P12_-L-F | CGGAATTCAAACATGCATAGCGTTTTCAATAGTTCGGTGTCG | | | | | | | | *Eco*RI | | | | | | | PCR for the *gltA*_P12_ left arm, *gltA*_P12_-L | |
| *^*^gltA*_P12_-L-R | CGGGATCCGGGCCTAGGGAAAGGATGATCTCGTA | | | | | | | | *Bam*HI | | | | | | |  |  |
| **Table S2** The oligonucleotides used in this study (Continued) | | | | | | | | | | | | | | | | | |
| Oligonucleotide | | | | 5’→3’ sequence ^a^ | | | | Cleavage sites | | | | | | | Purposes | | |
| *^*^gltA*_P12_-R-F | CGGGATCCGTTTCTCGAGTGGGCCGAACAAATATGTTTGAAAGGAT | | | | | | | | | *Bam*HI | | | | | PCR for the *gltA*_P12_ right arm, *gltA*_P12_-R | | |
| *^*^gltA*_P12_-R-R | CCCAAGCTTGCATGAACTGGGACTTGAAGTCCTC | | | | | | | | | *Hin*dIII | | | | |  |  |  |
| *gltA*_Up_-F | CGGAATTCTCGCGGTGGGAAACAAGCCAG | | | | | | | | | *Eco*RI | | | | | PCR for the *gltA* up segment, *gltA*_Up_ | | |
| *gltA*_Up_-P*_dapA_*-R | | **GCTACCTGCAGCTTTCTTAAACATTCTACC**CTCTTCCGGATTACGGAAGTAG | | | | | | | | *-* | | | | |  |  |  |
| P*dapA_gltA_*-F | **GGTAGAATGTTTAAGAAAGCTGCAGGTAGC** | | | | | | | | | ***-*** | | | | | PCR for the *dapA-L1* promoter, P_dapA-L1_ used for *gltA* modification | | |
| P*dapA_gltA_*-R | **CAGTAGCCACGATATCCCTTTCAAACAT**AGAGTTCAAGGTTACCTTC | | | | | | | | | | *-* | | | |  |  |  |
| *^**^ramA*-L-F | CCCAAGCTTGGGTACACTGTACCCTTGTC | | | | | | | | | *Hin*dIII | | | | | PCR for the *ramA* left arm, *ramA*-L | | |
| *^**^ramA*-L-R | CGCGGATCCCGCATCAGGAACGCCATTGC | | | | | | | | | *Bam*HI | | | | |  |  |  |
| *^**^ramA*-R-F | CGGGATCCGCGGGTTTTCATCTTTTTCCG | | | | | | | | | *Bam*HI | | | | | PCR for the *ramA* right arm, *ramA*-R | | |
| *^**^ramA*-R-R | CCGGAATTCCGAAGATCTATACGCGAACC | | | | | | | | | *Eco*RI | | | | |  |  |  |
| *ramA-*F | GTGGATACCCAGCGGATTAAAG | | | | | | | | | *-* | | | | | Verifying the ∆*ramA* deletion | | |
| *ramA-*R | TTAAGGCAGTGCGCCGATC | | | | | | | | | *-* | | | | |  |  |  |
| *prpC1*-L-F | CGGAATTCCGGCTTCGAAGGCGTCTACG | | | | | | | | | *Eco*RI | | | | | PCR for the *prpC1* left arm, *prpC1*-L | | |
| *prpC1*-L-R: | GCTCTAGATGGTGGGCAGCTCCCCAAAC | | | | | | | | | *Xba*I | | | | |  |  |  |
| *prpC1-*R-F: | GCTCTAGAGCCCTGCCTATTACATGCTG | | | | | | | | | *Xba*I | | | | | PCR for the *prpC1* right arm, *prpC1*-R | | |
| *prpC1*-R-R: | CCCAAGCTTGCTACGTTCCACTTCTCGCG | | | | | | | | | *Hin*dIII | | | | |  |  |  |
| *prpC1*-F | ATGAGTGACAGCCAAGTC | | | | | | | | | *-* | | | | | Verifying the ∆*prpC1* deletion | | |
| *prpC1*-R: | TTAGGTTCTCTCCGAAATC | | | | | | | | | *-* | | | | |  |  |  |
| *prpC2*-L-F | CGGAATTCCAGGAAGCCGGATTTGAAGGC | | | | | | | | | *Eco*RI | | | | | PCR for the *prpC2* left arm, *prpC2*-L | | |
| *prpC2*-L-R | CGGGATCCTCCACCAAATCTTCCACCGCG | | | | | | | | | *Bam*HI | | | | |  |  |  |
| *prpC2*-R-F | ACGCGTCGACTGCCTACCACCTGCTCGG | | | | | | | | | *Sal*I | | | | | PCR for the *prpC2* right arm *prpC2*-R | | |
| *prpC2*-R-R | CCCAAGCTTCATGCCCATTCCTGAGAC | | | | | | | | | *Hin*dIII | | | | |  |  |  |
| *prpC2-*F | GTGAATCACCATGTCCAGC | | | | | | | | | - | | | | | Verifying the ∆*prpC2* deletion | | |
| *prpC2-*R | TTAGCGCTTTTCAATGGGC | | | | | | | | | - | | | | |  |  |  |
| *prpR-*F | GCTCTAGAACATCGAGTTGATCTCTGAG | | | | | | | | | *Xba*I | | | | | PCR for the *prpR* segment | | |
| *prpR-*R | ACGCGTCGACTTAGTTGAGGAGTTGGACAG | | | | | | | | | *Sal*I | | | | |  |  |  |
| *MR-*F | CTCGCGAACAGACG***A***CACTTGTCCTTTGTG | | | | | | | | | - | | | | | Mutating G→A at loci 977 in *prpR* gene | | |
| *MR-*R | CACAAAGGACAAGTG***T***CGTCTGTTCGCGAG | | | | | | | | | - | | | | |  |  |  |
| *gdh-*F | **ATGACAGTTGATGAGCAGGTCTCTAAC** | | | | | | | | | - | | | | | PCR for the *gdh* operon | | |
| *gdh-*R | CCCAAGCTTTTAGATGACGCCCTGTGCCAG | | | | | | | | | *Hin*dIII | | | | |  |  |  |
| *gdh*_Up_-F | CGGAATTCTCGCGGTGGGAAACAAGCCAG | | | | | | | | | *Eco*RI | | | | | PCR for the *gdh* up segment, *gdh*_Up_ | | |
| *gdh*_Up_-P_dapA_-R | | **GCTACCTGCAGCTTTCTTAAACATTCTACC**GATTTCCTCGTTCCCATCTCGGC | | | | | | | | *-* | | PCR for the *gdh*_Up_ with the P_dapA-L1_ homologous sequence | | | | | |
| *gdh*_Up_-P_tac/M_-R | | **GTTATTTTGACAGACGAATGTATTTGTCA**GATTTCCTCGTTCCCATCTCGGC | | | | | | | | *-* | | PCR for the *gdh*_Up_ with the P_tac/M_ homologous sequence | | | | | |
| *gdh*_Up_-P_tuf_-R | | **CACTTACCCTACGCGCCTACTGACACGCT**GATTTCCTCGTTCCCATCTCGGC | | | | | | | | *-* | | PCR for the *gdh*_Up_ with the P_tuf_ homologous sequence | | | | | |
| *gdh*_Up_-P_sod_-R | | **GTGGCAGGCATCCTGTTTTAGAAAATC**ATTTCCTCGTTCCCATCTCGGC | | | | | | | | *-* | | PCR for the *gdh*_Up_ with the P_sod_ homologous sequence | | | | | |
| **Table S2** The oligonucleotides used in this study (Continued) | | | | | | | | | | | | | | | | | |
| Oligonucleotide | | | | 5’→3’ sequence ^a^ | | | Cleavage sites | | | | | | | Purposes | | | |
| P*dapA_gdh_*-F | **GGTAGAATGTTTAAGAAAGCTGCAGGTAGC** | | | | | | | ***-*** | | | | | PCR for the *dapA-L1* promoter, P_dapA-L1_ used for *gdh* modification | | | | |
| P*dapA_gdh_*-R | **GTTAGAGACCTGCTCATCAACTGTCAT**AGAGTTCAAGGTTACCTTC | | | | | | | *-* | | | | |  |  |  |  |  |
| P*tac/M_gdh_*-F | **TGACAAATACATTCGTCTGTCAAAATAAC** | | | | | | | *-* | | | | | PCR for the *tac*/*tac-M* promoter, P_tac_/P_tac-M_ used for *gdh* modification | | | | |
| P*tac/M_gdh_*-R | **GTTAGAGACCTGCTCATCAACTGTCAT**AAGACAAAGGACACACTTTAAG | | | | | | | *-* | | | | |  |  |  |  |  |
| P*tuf_gdh_*-F | **AGCGTGTCAGTAGGCGCGTAGGGTAAGTG** | | | | | | | *-* | | | | | PCR for the *tuf* promoter, P_tuf_ used for *gdh* modification | | | | |
| P*tuf_gdh_*-R | **GTTAGAGACCTGCTCATCAACTGTCAT**TGTATGTCCTCCTGGAG | | | | | | | *-* | | | | |  |  |  |  |  |
| P*sod_gdh_*-F | **GATTTTCTAAAACAGGATGCCTGCCAC** | | | | | | | - | | | | | PCR for the *sod* promoter, P_sod_ used for *gdh* modification | | | | |
| P*sod_gdh_*-R | **GTTAGAGACCTGCTCATCAACTGTCAT**GGGTAAAAAATCCTTTCGTAC | | | | | | | - | | | | |  |  |  |  |  |
| *ppc-*F^r^ | GAACTGGTCGAACAAGCGC | | | | | | | - | | | | | qRT-PCR for *ppc* | | | | |
| *ppc-*R^r^ | GCCCTCATTGAGTTTCAGC | | | | | | | - | | | | |  |  |  |  |  |
| *pyc-*F^r^ | CCGACTTTGAAACTGCTG | | | | | | | - | | | | | qRT-PCR for *pyc* | | | | |
| *pyc*-R^r^ | CACACCATGAGGCTTGTTC | | | | | | | - | | | | |  |  |  |  |  |
| *aceE-*F^r^ | CGAGTGGATGGATTCACTCG | | | | | | | - | | | | | qRT-PCR for *aceE* | | | | |
| *aceE-*R^r^ | CATGATGGCTGCGTTCCAG | | | | | | | - | | | | |  |  |  |  |  |
| *^***^gltA*-F^r^ | TGTTTGAAAGGGATATCGTG | | | | | | | - | | | | | qRT-PCR for *gltA* | | | | |
| *^***^gltA-*R^r^ | AGTCTCAGACAGCATCTTGC | | | | | | | - | | | | |  |  |  |  |  |
| *^***^icd*-F^r^ | AAGCACCGTTGCTCGCGACCTA | | | | | | | - | | | | | qRT-PCR for *icd* | | | | |
| *^***^icd-*R^r^ | CGGCCAGCCAGTGAAATGTC | | | | | | | - | | | | |  |  |  |  |  |
| *^***^aceA*-F^r^ | ATGTCAAACGTTGGAAAGCC | | | | | | | - | | | | | qRT-PCR for *aceA* | | | | |
| *^***^aceA-*R^r^ | TGTGCTCCTCGATGACGGAA | | | | | | | - | | | | |  |  |  |  |  |
| *^***^aceB*-F^r^ | TGACTGAACAGGAACTGTTGTC | | | | | | | - | | | | | qRT-PCR for *aceB* | | | | |
| *^***^aceB-*R^r^ | AGGGAGTACCGCTTCGGTTA | | | | | | | - | | | | |  |  |  |  |  |
| *^***^fumA*-F^r^ | ATGACCGAGCAGGAATTCCG | | | | | | | - | | | | | qRT-PCR for *fumA* | | | | |
| *^***^fumA-*R^r^ | GATTCCAGACCACGACCAGA | | | | | | | - | | | | |  |  |  |  |  |
| *^***^mdh*-F^r^ | ATGAATTCCCCGCAGAACGT | | | | | | | - | | | | | qRT-PCR for *mdh* | | | | |
| *^***^mdh-*R^r^ | TCAGCTCAACAGGGGTTTCA | | | | | | | - | | | | |  |  |  |  |  |
| *^***^mqo*-F^r^ | ATGTCAGATTCCCCGAAGAA | | | | | | | - | | | | | qRT-PCR for *mqo* | | | | |
| *^***^mqo-*R^r^ | AGACGATCTGAGTCCAGCTT | | | | | | | - | | | | |  |  |  |  |  |
| *^****^malE-*F^r^ | GCAAGGCTCAGCTGTTTAGCTC | | | | | | | - | | | | | qRT-PCR for *malE* | | | | |
| *^****^malE-*R^r^ | GCATATCTACAGCTGCAACGC | | | | | | | - | | | | |  |  |  |  |  |
| *^****^apsC-*F^r^ | CTGTGCCAATGAATGAAGAC | | | | | | | - | | | | | qRT-PCR for *aspC* | | | | |
| *^****^aspC-*R^r^ | GTGAGTGATCTTCGAAGTA | | | | | | | - | | | | |  |  |  |  |  |
| *sdhA-*F^r^ | CTCGTCGTTGGCACCGGC | | | | | | | - | | | | | qRT-PCR for *sdhA* | | | | |
| *sdhA-*R^r^ | CGCGACCACGGTAGTCGC | | | | | | | - | | | | |  |  |  |  |  |
| *sdhB-*F^r^ | GACGCCGTCGCGCAGCTG | | | | | | | - | | | | | qRT-PCR for *sdhA* | | | | |
| *sdhB-*R^r^ | GTCGCCTTCCTTGTAGCTG | | | | | | | - | | | | |  |  |  |  |  |
| *gdh-*F^r^ | GTCAGCTCATCTTCCGTG | | | | | | | - | | | | | qRT-PCR for *gdh* | | | | |
| *gdh-*R^r^ | GATGTGGCGGTGTAGCTC | | | | | | | - | | | | |  |  |  |  |  |
| **Table S2** The oligonucleotides used in this study (Continue) | | | | | | | | | | | | | | | | | |
| Oligonucleotide | | | | 5’→3’ sequence ^a^ | | Cleavage sites | | | | | | | | Purposes | | | |
| 16S-F^r^ | ACCTGGAGAAGAAGCACCG | | | | | - | | | | | | | | qRT-PCR for 16S rRNA | | | |
| 16S-R^r^ | TCAAGTTATGCCCGTATCG | | | | | - | | | | | | | |  |  |  |  |

^a^ Cleavage sites are underlined; Nucleotide in bold and italic: mutation site; Sequence in italic: SD sequences [[24](#_ENREF_24)]; Sequence in bold: homologous recombination sequences; ^r^: The premiers for qRT-PCR; *^*^*: The sequence refers to [van Ooyen J, Emer D, Bussmann M, Bott M, Eikmanns BJ and Eggeling L [25]](#_ENREF_25), *^**^*: The sequence refers to [Cramer A, Gerstmeir R, Schaffer S, Bott M and Eikmanns BJ [26]](#_ENREF_26) and *^*^*^*^*^*^*: The sequence refers to [Han SO, Inui M and Yukawa H [27]](#_ENREF_27); -: No cleavage sites.

**Table S3** The specific activities of enzymes in genetically modified *C. glutamicum* strains and original strain *C. glutamicum* JL-6 as well as wild-type strain *C. glutamicum* ATCC13032.

| *C. glutamicum* strains |  | | Specific activity of (U mg^-1^ protein) ^a^ | | | | | | |
| --- | --- | --- | --- | --- | --- | --- | --- | --- | --- |
|  | PK | PEPCx | | PEPCk | PCx ^b^ | ODx | MQO | MDH | MalE |
| ATCC13032 | 1.31±0.13 | 0.37±0.05 | | 0.13±0.04 | 0.04±0.02 | 0.67±0.14 | 0.12±0.05 | 0.09±0.01 | 3.66±0.27 |
| JL-6 | 1.44±0.18 | 0.29±0.02 | | 0.10±0.06 | 0.07±0.05 | 0.46±0.05 | 0.09±0.03 | 0.07±0.00 | 2.18±0.24 |
| JL-61 | 1.43±0.23 | 0.30±0.07 | | < 0.01 | 0.08±0.03 | 0.47±0.10 | 0.09±0.01 | 0.08±0.01 | 2.18±0.15 |
| JL-62 | 1.46±0.21 | 0.29±0.11 | | 0.12±0.11 | 0.05±0.04 | < 0.01 | 0.08±0.04 | 0.08±0.02 | 2.20±0.21 |
| JL-63 | 1.44±0.05 | 0.28±0.05 | | 0.13±0.05 | 0.07±0.07 | 0.48±0.04 | 0.09±0.02 | 0.07±0.02 | < 0.01 |
| JL-64 | 1.40±0.14 | 0.30±0.03 | | 0.09±0.08 | 0.09±0.06 | 0.45±0.06 | 0.07±0.03 | 0.09±0.01 | 5.07±0.44 |
| JL-65 | 1.48±0.11 | 0.26±0.08 | | < 0.01 | 0.05±0.04 | < 0.01 | 0.08±0.01 | 0.08±0.03 | 2.21±0.21 |
| JL-66 | 1.29±0.16 | 0.84±0.16 | | < 0.01 | 0.04±0.01 | 0.53±0.12 | 0.08±0.03 | 0.08±0.01 | 2.17±0.19 |
| JL-67 | 1.60±0.24 | 0.21±0.04 | | 0.14±0.07 | 0.25±0.05 | < 0.01 | 0.07±0.02 | 0.08±0.02 | 2.17±0.06 |
| JL-68 | 1.52±0.13 | 0.55±0.13 | | < 0.01 | 0.21±0.08 | < 0.01 | 0.07±0.04 | 0.09±0.03 | 2.03±0.22 |

^a^ PK: Pyruvate kinase, PEPCx: Phosphoenolpyruvate carboxylase, PEPCk: Phosphoenolpyruvate carboxykinase, PCx: Pyruvate carboxylase, ODx: Oxaloacetate decarboxylase, MQO: Malate:quinone oxidoreductase, MDH: Malate dehydrogenase; MalE: Malic enzyme; and the corresponding enzymes assay was performed as previously described.

^b^ The activity unit of PCx is U (mg DCW)^-1^.

All data are meaning values of three determinations of three independent experiments with ± standard deviation (SD).

**Table S4** L-lysine production, by-product accumulation, DCW, maximal specific growth rate (μ_max._), maximal specific production rate of L-lysine (*q*_Lys, max._) and glucose conversion efficiency (α) of modified PEP-pyruvate-OAA node *C. glutamicum* strains.

| *C. glutamicum*  strains | | Concentration of (g L^-1^) ^a^ | | | | | | | | DCW  (g L^-1^) | μ_max._  (h^-1^) | *q*_Lys, max._  (g g^-1^ h^-1^) | α (%) ^b^ |
| --- | --- | --- | --- | --- | --- | --- | --- | --- | --- | --- | --- | --- | --- |
|  |  | L-Lys | L-Asp | L-Ala | L-Val | PEP | Pyr | OAA | Mal |  |  |  |  |
| WT | < 0.01 | | < 0.01 | 1.02±0.07 | < 0.01 | < 0.01 | < 0.01 | < 0.01 | < 0.01 | 21.07±1.76 | 0.37 | 0.00 | 0.00 |
| JL-6 | 14.47±0.52 | | < 0.01 | 5.98±0.33 | 1.28±0.15 | 0.13±0.02 | 0.16±0.01 | < 0.01 | < 0.01 | 11.24±1.05 | 0.22 | 0.25±0.01 | 36.18 |
| JL-61 | 14.53±1.07 | | < 0.01 | 5.44±0.42 | 1.23±0.03 | 0.09±0.04 | 0.20±0.03 | < 0.01 | < 0.01 | 11.24±0.67 | 0.23 | 0.23±0.05 | 36.33 |
| JL-62 | 14.60±0.26 | | < 0.01 | 5.05±0.27 | 1.19±0.18 | 0.11±0.02 | 0.15±0.03 | < 0.01 | < 0.01 | 11.23±1.12 | 0.21 | 0.26±0.03 | 36.50 |
| JL-63 | 14.51±0.97 | | < 0.01 | 5.87±0.51 | 1.27±0.12 | 0.13±0.03 | 0.17±0.03 | < 0.01 | < 0.01 | 11.30±0.96 | 0.24 | 0.24±0.02 | 36.28 |
| JL-64 | 14.21±0.52 | | < 0.01 | 6.03±0.26 | 1.28±0.06 | 0.14±0.01 | 0.12±0.02 | < 0.01 | < 0.01 | 11.43±1.03 | 0.22 | 0.22±0.03 | 35.53 |
| JL-65 | 15.34±0.61 | | < 0.01 | 4.48±0.19 | 1.11±0.05 | 0.08±0.02 | 0.18±0.03 | < 0.01 | < 0.01 | 10.54±0.76 | 0.22 | 0.27±0.05 | 38.35 |

^a^ L-Lys: L-lysine; L-Asp: L-aspartate; L-Ala: L-alanine; L-Val: L-valine; PEP: Phosphoenolpyruvate; Pyr: Pyruvate; OAA: Oxaloacetate; Mal: Malate.

^b^ α: The rate of glucose conversion into L-lysine.

All data are meaning values of three determinations of three independent experiments with ± SD.

**Table S5** L-lysine production, by-product accumulation, DCW, maximal specific growth rate (μ_max._), maximal specific production rate of L-lysine (*q*_Lys, max._) and glucose conversion efficiency (α) of genetically defined *C. glutamicum* strains during modified the glutamate dehydrogenase of *C. glutamicum* JL-68∆*gltA*∆*prpC1prpR*^G977A^ (or *C. glutamicum* JL-610).

| *C. glutamicum* strains | Concentration of (g L^-1^) ^a^ | | | | | DCW | μ_max_ (h^-1^) | *q*_Lys, max._  (g g^-1^ h^-1^) | α (%) |
| --- | --- | --- | --- | --- | --- | --- | --- | --- | --- |
|  | L-Lys | L-Asp | L-Glu | α-KG | OAA |  |  |  |  |
| JL-610 | 20.18±3.61 | < 0.01 | 1.04±0.15 | 0.54±0.11 | 1.21±0.04 | 6.03±1.12 | 0.14 | 0.58±0.33 | 50.45 |
| JL-610P_dapA-L1_ *gdh* | 9.12±0.87 | < 0.01 | 0.19±0.02 | 1.05±0.20 | 1.32±0.22 | 5.98±1.01 | 0.15 | 0.41±0.03 | 22.80 |
| JL-610P_tac_ *gdh* | 21.23±1.23 | < 0.01 | 2.51±0.22 | 0.58±0.16 | 1.03±0.11 | 5.84±1.35 | 0.14 | 0.63±0.08 | 53.08 |
| JL-610P_tac-M_ *gdh* | 20.71±2.16 | < 0.01 | 2.27±0.14 | 0.28±0.07 | 0.25±0.07 | 5.36±1.07 | 0.13 | 0.67±0.05 | 51.78 |
| JL-610P_tuf_ *gdh* | 10.12±1.13 | < 0.01 | 0.72±0.12 | 0.15±0.01 | < 0.01 | 4.71±0.95 | 0.10 | 0.62±0.13 | 25.33 |
| JL-610P_sod_ *gdh* | 18.34±1.77 | < 0.01 | 0.85±0.10 | 0.22±0.09 | < 0.01 | 5.14±0.72 | 0.13 | 0.74±0.11 | 45.85 |

^a^ L-Lys: L-lysine; L-Asp: L-aspartate; L-Glu: L-glutamate; α-KG: α-ketoglutarate; OAA: Oxaloacetate.

All data are meaning values of three determinations of three independent experiments with ± SD.

**Fig. S1.** SDS-PAGE analyzes the PEPCx and PCx expression in *C. glutamicum*. *Lane 1* *C. glutamicum* JL-67 without added IPTG; *Lane 2* *C. glutamicum* JL-67 with added IPTG; *Lane 3* *C. glutamicum* JL-66 without added IPTG; *Lane 4* *C. glutamicum* JL-66 with added IPTG; *Lane 5* *C. glutamicum* JL-68 without added IPTG; *Lane 6* *C. glutamicum* JL-68 with added IPTG; *Lane M* the protein marker.

**Fig. S2.** The cell growth and L-lysine production of *C. glutamicum* JL-6 and its genetically modified strains in shake-flask culture (a) as well as their growing performance on CgXIIM medium plates containing 5 g L^-1^ glucose (b-1) and supplemented with 1 g L^-1^ potassium acetate (b-2). i-iv indicates strain JL-6, JL-6 ∆*aceE*, JL-6 *icd*^A1G^, JL-6 ∆*aceE* *icd*^A1G^, respectively.

**Fig. S3.** Comparison of by-products accumulation including organic acids (a) and amino acids (b) of strain JL-68 and its CS-leaky or deficient strains in shake-flasks culture with CgXIIM minimal medium containing 40 g L^-1^ glucose. In Fig. b, the data in Fig. 3b-1 refers the left ordinate values, whereas the data in Fig. 3b-2 refers the right ordinate values. The data represent mean values and standard deviations obtained from three independent cultivations. Abbreviation: *PEP* Phosphoenolpyruvate, *Pyr* Pyruvate, *Lac* Lactate, *Ac-CoA* Acetyl coenzyme A, *Ac* Acetic acid, *α-KG* αketoglutarate, *OAA* Oxaloacetate, *Mal* Malate, *Ala* L-alanine, *Val* L-valine, *Leu* L-leucine, *Glu* L-glutamate, *Asp* L-aspartate, *Tyr* L-tyrosine, *Phe* L-phenylalanine, *Trp* L-tryptophan.

**Fig. S4.** Comparison of by-products accumulation including organic acids (a) and amino acids (b) of strain JL-69 and its genetically modified strains in shake-flasks culture with CgXIIM minimal medium containing 40 g L^-1^ glucose. The data represent mean values and standard deviations obtained from three independent cultivations. Abbreviation: *PEP* Phosphoenolpyruvate, *Pyr* Pyruvate, *Lac* Lactate, *Ac-CoA* Acetyl coenzyme A, *Ac* Acetic acid, *Mal* Malate, *Ala* L-alanine, *Val* L-valine, *Leu* L-leucine, *Tyr* L-tyrosine, *Phe* L-phenylalanine, *Trp* L-tryptophan.

**Fig. S5.** The relative expression level of genes involved in central carbon metabolism in strain JL-69P_tac-M_ *gdh* between addition of 0.2 mg L^-1^ (white bars) and of 0.6 mg L^-1^ biotin (gray bars). The standard errors are shown as bars

**Fig. S6.** Strategy used for construction of recombinant plasmid used for the replacement of promoter of the target gene. *T* represents the modified gene; *T_Up_* represents the upstream regions of the modified gene; *P* represents the promoter; *P1-P6* represents the primers; *E1* and *E2* represent the restriction enzymes. The lines in the same color represent the homologous sequence.

**Supplementary References**

1. Xu JZ, Han M, Ren XD, Zhang WG: **Modification of aspartokinase III and dihydrodipicolinate synthetase increases the production of L-lysine in *Escherichia coli*.** *Biochem Eng J* 2016, **114:**82-89.

2. van der Rest ME, Lange C, Molenaar D: **A heat shock following electroporation induces highly efficient transformation of *Corynebacterium glutamicum* with xenogeneic plasmid DNA.** *Appl Microbiol Biot* 1999, **52:**541-545.

3. Xu JZ, Han M, Zhang JL, Guo YF, Qian H, Zhang WG: **Improvement of L-lysine production combines with minimization of by-products synthesis in *Corynebacterium glutamicum*.** *J Chem Technol Biot* 2014, **89:**1924-1933.

4. Xu JZ, Han M, Zhang JL, Guo YF, Zhang WG: **Metabolic engineering *Corynebacterium glutamicum* for the L-lysine production by increasing the flux into L-lysine biosynthetic pathway.** *Amino Acids* 2014, **46:**2165-2175.

5. Becker J, Zelder O, Hafner S, Schroder H, Wittmann C: **From zero to hero-Design-based systems metabolic engineering of *Corynebacterium glutamicum* for L-lysine production.** *Metab Eng* 2011, **13:**159-168.

6. Xu JZ, Zhang JL, Hu KH, Zhang WG: **The relationship between lignin peroxidase and manganese peroxidase production capacities and cultivation periods of mushrooms.** *Microbial Biotechnol* 2013, **6:**241-247.

7. Claes WA, Puhler A, Kalinowski J: **Identification of two *prpDBC* gene clusters in *Corynebacterium glutamicum* and their involvement in propionate degradation via the 2-methylcitrate cycle.** *J Bacteriol* 2002, **184:**2728-2739.

8. Xu JZ, Zhang JL, Guo YF, Zhang WG: **Genetically modifying aspartate aminotransferase and aspartate ammonia-lyase affects metabolite accumulation in L-lysine producing strain derived from *Corynebacterium glutamicum* ATCC13032.** *J Mol Catal B-Enzym* 2015, **113:**82-89.

9. Marx A, Eikmanns BJ, Sahm H, de Graaf AA, Eggeling L: **Response of the central metabolism in *Corynebacterium glutamicum* to the use of an NADH-dependent glutamate dehydrogenase.** *Metabolic Engineering* 1999, **1:**35-48.

10. Jetten MSM, Gubler ME, Lee SH, Sinskey AJ: **Structural and functional analysis of pyruvate kinase from *Corynebacterium glutamicum*.** *Appl Environ Microbiol* 1994, **60:**2501-2507.

11. Mitsuhashi S, Hayashi M, Ohnishi J, Ikeda M: **Disruption of malate : quinone oxidoreductase increases L-lysine production by *Corynebacterium glutamicum*.** *Biosci Biot Biochem* 2006, **70:**2803-2806.

12. Krause JP, Polen T, Youn JW, Emer D, Eikmanns BJ, Wendisch VF: **Regulation of the malic enzyme gene *malE* by the transcriptional regulator MalR in *Corynebacterium glutamicum*.** *J Biotechnol* 2012, **159:**204-215.

13. Becker J, Klopprogge C, Wittmann C: **Metabolic responses to pyruvate kinase deletion in lysine producing *Corynebacterium glutamicum*.** *Microb Cell Fact* 2008, **7:**8.

14. Gourdon P, Baucher MF, Lindley ND, Guyonvarch A: **Cloning of the malic enzyme gene from *Corynebacterium glutamicum* and role of the enzyme in lactate metabolism.** *Appl Environ Microbiol* 2000, **66:**2981-2987.

15. Sauer U, Eikmanns BJ: **The PEP-pyruvate-oxaloacetate node as the switch point for carbon flux distribution in bacteria.** *FEMS Microbiol Rev* 2005, **29:**765-794.

16. Ohnishi J, Hayashi M, Mitsuhashi S, Ikeda M: **Efficient 40 ^o^C fermentation of L-lysine by a new *Corynebacterium glutamicum* mutant developed by genome breeding.** *Appl Microbiol Biotechnol* 2003, **62:**69-75.

17. Neuner A, Heinzle E: **Mixed glucose and lactate uptake by *Corynebacterium glutamicum* through metabolic engineering.** *Biotechnol J* 2011, **6:**318-329.

18. Netzer R, Krause M, Rittmann D, Peters-Wendisch PG, Eggeling L, Wendisch VF, Sahm H: **Roles of pyruvate kinase and malic enzyme in *Corynebacterium glutamicum* for growth on carbon sources requiring gluconeogenesis.** *Arch Microbiol* 2004, **182:**354-363.

19. Georgi T, Rittmann D, Wendisch VF: **Lysine and glutamate production by *Corynebacterium glutamicum* on glucose, fructose and sucrose: roles of malic enzyme and fructose-1,6-bisphosphatase.** *Metab Eng* 2005, **7:**291-301.

20. Xu DQ, Tan YZ, Shi F, Wang XY: **An improved shuttle vector constructed for metabolic engineering research in *Corynebacterium glutamicum*.** *Plasmid* 2010, **64:**85-91.

21. Xu DQ, Tan YZ, Li Y, Wang XY: **Construction of a novel promoter-probe vector and its application for screening strong promoter for *Brevibacterium flavum* metabolic engineering.** *World J Microbiol Biot* 2011, **27:**961-968.

22. Schafer A, Tauch A, Jager W, Kalinowski J, Thierbach G, Puhler A: **Small mobilizable multipurpose cloning vectors derived from the Escherichia coli plasmids pK18 and pK19 - selection of defined deletions in the chromosome of *Corynebacterium glutamicum*.** *Gene* 1994, **145:**69-73.

23. Xu JZ, Zhang JL, Han M, Zhang WG: **A method for simultaneous gene overexpression and inactivation in the *Corynebacterium glutamicum* genome.** *J Ind Microbiol& Biot* 2016, **43:**1417-1427.

24. Srivastava P, Deb JK: **Gene expression systems in corynebacteria.** *Protein Express Purif* 2005, **40:**221-229.

25. van Ooyen J, Emer D, Bussmann M, Bott M, Eikmanns BJ, Eggeling L: **Citrate synthase in *Corynebacterium glutamicum* is encoded by two *gltA* transcripts which are controlled by RamA, RamB, and GlxR.** *J Biotechnol* 2011, **154:**140-148.

26. Cramer A, Gerstmeir R, Schaffer S, Bott M, Eikmanns BJ: **Identification of RamA, a novel LuxR-type transcriptional regulator of genes involved in acetate metabolism of *Corynebacterium glutamicum.*** *J Bacteriol* 2006, **188:**2554-2567.

27. Han SO, Inui M, Yukawa H: **Transcription of *Corynebacterium glutamicum* genes involved in tricarboxylic acid cycle and glyoxylate cycle.** *J Mol Microbiol Biot* 2008, **15:**264-276.
